# Dietary pattern and health-related quality of life among breast cancer survivors

**DOI:** 10.1186/s12905-018-0555-7

**Published:** 2018-05-10

**Authors:** Na-Hui Kim, Sihan Song, So-Youn Jung, Eunsook Lee, Zisun Kim, Hyeong-Gon Moon, Dong-Young Noh, Jung Eun Lee

**Affiliations:** 1 Department of Food and Nutrition, Sookmyung Women’s University, Cheongpa-ro 47-gil 100, Yongsan-gu, Seoul, 04310 Korea; 2 Department of Food and Nutrition, Seoul National University, 1 Gwanak-ro, Gwanak-gu, Seoul, 08826 Korea; 3 Center for Breast Cancer, Research Institute and Hospital, National Cancer Center, 323 Ilsan-ro, Ilsandong-gu, Goyang-si, Gyeonggi-do 10408 Korea; 4 Department of Surgery, Soonchunhyang University College of Medicine, 1174 Joong-dong, Wonmi-gu, Bucheon, Gyeonggi-do 14584 Korea; 5 Department of Surgery and Cancer Research Institute, Seoul National University College of Medicine, 101 Daehakro, Jongno-gu, Seoul, 03080 Korea; 6 Breast Care Center, Seoul National University Hospital, 101 Daehakro, Jongno-gu, Seoul, 03080 Korea

**Keywords:** Breast cancer, Breast cancer survivors, Dietary patterns, Health-related quality of life

## Abstract

**Background:**

There is limited evidence for the association between dietary pattern and quality of life among breast cancer survivors. We examined the association between dietary patterns and health-related quality of life (HRQoL) among Korean breast cancer survivors.

**Methods:**

Our study included a total of 232 women, aged 21 to 79 years, who had been diagnosed with stage I to III breast cancer and who underwent breast cancer surgery at least 6 months prior to our baseline evaluation. We assessed HRQoL using the European Organization for Research and Treatment of Cancer Quality of Life Questionnaire Core 30 (EORTC QLQ-C30) and the Quality of Life Questionnaire Breast Cancer Module 23 (QLQ-BR23). We conducted a factor analysis to identify the major dietary patterns and used a generalized linear model to obtain the least squares mean (LS mean) and 95% confidence interval (CI) for HRQoL according to the dietary pattern scores.

**Results:**

We identified 2 major dietary patterns: the Healthy dietary pattern and the Western dietary pattern. We found that breast cancer survivors who had higher Healthy dietary pattern scores tended to have lower dyspnea scores but higher insomnia scores, compared to breast cancer survivors with lower Healthy dietary pattern scores. For dyspnea, the LS mean (95% CI) was 8.86 (5.05-15.52) in the bottom quartile and 2.87 (1.62-5.08) in the top quartile (*p* for trend = 0.005). This association was limited to survivors with stage I for dyspnea or survivors with stage II or III for insomnia.

**Conclusions:**

Healthy dietary patterns were associated with better scores for dyspnea but worse scores for insomnia among breast cancer survivors. Other components of EORTC QLQ did not vary by dietary patterns overall, but they warrant further investigation for subgroups of breast cancer survivors.

**Electronic supplementary material:**

The online version of this article (10.1186/s12905-018-0555-7) contains supplementary material, which is available to authorized users.

## Background

Breast cancer is the most common cancer among women worldwide [1]. In Korea, the incidence of breast cancer ranks second to thyroid cancer among women [2]. The age-standardized incidence rate of breast cancer has also steadily increased, reaching 45.7 per 100,000 in 2013. The five-year survival rate for Korean breast cancer patients has also improved remarkably from 78.0% in 1993-1995 to 91.5% in 2008-2013 [2].

The improvement in survival emphasizes the importance of supportive care, diet, and health-related quality of life (HRQoL) for breast cancer survivors. Dietary patterns address the effects of diet as a whole, rather than those of individual nutrients or foods, and may therefore enable investigators to examine associations with overall diet. Identifying healthy dietary patterns that lower the risk of poor health outcomes may be important to provide evidence-based dietary guidelines for breast cancer survivors. However, studies regarding diet after breast cancer diagnosis are lacking, and only a few studies have explored the association between dietary pattern and breast cancer prognosis [3–5]. Recent prospective cohort studies found that both Western and Prudent dietary patterns were associated with breast cancer prognosis. The Prudent dietary pattern was inversely associated with death from other causes, but Western dietary pattern was associated with an elevated risk of mortality from other causes than breast cancer [3]. Women with high Prudent dietary pattern scores had a significantly decreased risk of overall and other-causes of mortality, compared to women with low scores [4]. A prospective cohort study originating from a case-control study in Europe suggested that the Healthy dietary pattern was inversely associated with overall mortality and breast cancer recurrence among postmenopausal breast cancer survivors who had been diagnosed with stage I-IV breast cancer. In this same study, the unhealthy dietary pattern was associated with an increased risk of non-breast cancer-related death [5].

Several studies have examined the relationship of diet quality scores based on the recommended guidelines with breast cancer prognosis. In the Women’s Health Initiative’s Dietary Modification Trial and Observational Study, better post-diagnostic diet quality based on scores on the Healthy Eating Index (HEI)-2005 was associated with a reduced risk of death, particularly from non-breast cancer-related causes [6]. In the Health, Eating, Activity, and Lifestyle (HEAL) Study, increased dietary quality scores on the HEI-2005 were related to a lower risk of mortality from breast cancer [7]. The Nurses’ Health Study explored four dietary quality scores in relationship to total, breast cancer-specific, and non-breast cancer-related deaths. That study reported a lower risk of non-breast cancer-related deaths with high alternate HEI scores but a higher risk of breast cancer-specific deaths with high recommended food scores [8].

Breast cancer survivors’ quality of life as an outcome in relation to diet quality or dietary pattern has been examined in epidemiological studies. The HEAL study found that women with high diet quality scores on the HEI-2010 had lower fatigue scores, compared to women with low diet quality scores [9]. A higher ratio of ω-6 relative to ω-3 polyunsaturated fats was associated with greater fatigue [10]. The HEAL study also reported that breast cancer survivors with healthier diet quality had significantly better scores of mental health functioning and physical functioning measured approximately 10 months later [11]. Korean breast cancer patients with high adherence to the American Cancer Society (ACS)‘s Nutrition and Physical Activity Guidelines for Cancer Prevention [12] had higher scores of social functioning compared to those with low adherence [13].

We aimed to identify patterns of post-diagnosis diet and to examine whether dietary patterns were associated with HRQoL levels among Korean breast cancer survivors.

## Methods

### Participants

Breast cancer survivors were recruited between September 2012 and November 2015 from three large hospitals. A total of 286 patients, aged 21-79 years, who had been diagnosed with primary breast cancer were enrolled in our study. Among the study participants, we excluded patients who had been diagnosed with stage 0 breast cancer or had missing data in their medical records (*n* = 29), those with other cancers before the breast cancer diagnosis (*n* = 7) or those who had breast cancer surgery within 6 months before recruitment (*n* = 12). In addition, we excluded women who did not completely record their dietary data (*n* = 5) and who reported implausible energy intake (above the log-transformed mean ± 3 sd, n = 1). As a result, we included a total of 232 breast cancer survivors who had been diagnosed with primary breast cancer (stage I-III) more than 6 months after breast cancer surgery. All participants gave written informed consent at enrollment. This study was approved by the Institutional Review Board at Seoul National University Hospital, the National Cancer Center and Soonchunhyang University Hospital in Korea.

### Collection of sociodemographic, lifestyle and clinical information

A trained research nurse asked the participants about their demographic information, physical activity, alcohol consumption, smoking status, clinical information, parity, supplement use, health-related quality of life, family history, sun exposure and dietary principles for breast cancer survivors using a structured questionnaire. The score of physical activity was calculated as metabolic equivalent task (MET)-hour per week.

Through medical record data, we obtained information regarding weight and height at diagnosis of breast cancer and clinical information, including other incidences of cancer before their breast cancer diagnosis, the diagnosis date of primary breast cancer, menopausal status and menopausal hormone use at diagnosis, recurrence and metastasis after diagnosis, hormone receptor status, cancer stage, tumor size, date of surgery and initial treatment type. Body mass index (BMI, kg/m^2^) at diagnosis was calculated by dividing a participant’s weight (kg) by his/her height (m^2^).

### Assessment of dietary intake

Participants completed a non-consecutive 3-day dietary record on 2 weekdays and 1 weekend day. All foods and beverages that they consumed were recorded. To help with recording, we provided additional food photograph booklets originally designed by our study team. Dietary intake of nutrients and energy were calculated using the Computer-Aided Nutritional Analysis Program (CAN-Pro) version 4.0 (Korean Nutrition Information Center, Seoul, Korea). We grouped a total of 1150 food items one-by-one into 39 food groups based on the similarities of their nutrient profiles. Next, we assigned new food codes to all food items for analysis and combined the intake amount for each food group per person. Finally, we calculated the daily amount (g/day) of each food group.

### Assessment of health-related quality of life

We asked participants to answer the questions about HRQoL using a validated Korean version of the European Organization for Research and Treatment of Cancer Quality of Life Questionnaire Core 30 (EORTC QLQ-C30) version 3.0 and the Quality of Life Questionnaire Breast Cancer Module 23 (QLQ-BR23) [14, 15]. The 30-item QLQ-C30 is used to assess the HRQoL of cancer survivors and is composed of the following categories: (1) global health status/quality of life scale, (2) functional scales (physical, role, emotional, cognitive, and social) and (3) symptom scales (fatigue, nausea and vomiting, pain, dyspnea, insomnia, appetite loss, constipation, diarrhea, and financial difficulty). The QLQ-BR23, is composed of functioning and symptom scales and is categorized into the following domains: (1) functional scales (body image, sexual functioning, sexual enjoyment, and future perspective) and (2) symptom scales (systemic therapy side effects, breast symptoms, arm symptoms, and upset by hair loss). In our study, we did not included ‘sexual enjoyment’, because 70% of the participants did not respond to this scale. We transformed the raw scores of the 4-point or 7-point scales to a 0 to 100 scale based on the EORTC scoring manual. A higher score reflects a higher quality of life in functioning and global health status/quality of life and a lower quality of life in symptoms.

### Statistical analysis

We conducted a factor analysis to determine dietary patterns for breast cancer survivors. The dietary patterns were generated by a principal component analysis using an orthogonal rotation procedure [16]. We determined two factors based on eigenvalue, screening test, and our interpretations. We labeled these two factors as “Healthy dietary pattern” and “Western dietary pattern”. The factor score for each pattern was calculated by summing the intakes of all the food groups weighted by their factor loadings. Each dietary pattern score was categorized by quartiles for all participants. Individuals with high dietary pattern scores had a greater tendency to follow the pattern, compared to individuals with low scores. The association between HRQoL scores and each dietary pattern was estimated from the least squares mean (LS mean) and 95% confidence interval (95% CI) using a generalized linear model (GLM). In addition, we compared the top quartile with the bottom quartile of the factor scores using the contrast command. All models were adjusted for age at diagnosis (years; continuous), BMI (kg/m^2^; continuous) at diagnosis, energy intake (kcal/day; continuous), marital status (married/cohabitation and unmarried/divorced/widowed), education level (high school or below and college or above), breast cancer stage at diagnosis (I, II, and III), physical activity (MET-h/week; continuous), time since surgery (6 months- < 1 year, 1 year- < 5 years, and ≥ 5 years) and menopausal status at diagnosis (pre- or postmenopausal status). To test for trends across categories, we assigned the median value to the quartile category of dietary pattern being used as a continuous variable. If we had missing variables (the proportion for each < 1%), we assigned the participants to the median or the common category. We used the log-transformed variables of the HRQoL scores to meet normality, and these variables were exponentiated. If there was no information (unknown) regarding menopausal status at diagnosis in the medical records, we regarded the participant as postmenopausal at diagnosis if their age at menopause on the questionnaire was younger than the age at diagnosis. We regarded the participant as premenopausal at diagnosis if their age at menopause on the questionnaire was older than the age at diagnosis. When information regarding age at menopause was missing (*n* = 10), we considered the participant postmenopausal if they had been diagnosed after the age of 50, which was the median age of menopause in Korean women in 2013 [17]. We conducted a subgroup analysis by stage (I and II or III), menopausal status at diagnosis (pre or postmenopausal), and time since surgery (< 2 or ≥ 2 years). All statistical analyses were performed using SAS software 9.4 (SAS Institute Inc., Cary, NC). Statistical significance was defined as a *P*-value < 0.05 in a two-sided test. The resulting raw *p*-values were adjusted for multiple comparisons using the False Discovery Rate (FDR) method [18].

## Results

### Factors identified for dietary patterns

We identified two dietary patterns: “Healthy dietary pattern” and “Western dietary pattern”. We presented a factor loading of more than 0.20 or less than − 0.20 (Table 1). The Healthy dietary pattern explained 2.88% of the variance, and the Western dietary pattern explained 2.01%. The Healthy dietary pattern was characterized by higher intake of vegetables, whole grains, soy, potatoes, fish, fruits, yogurt, kimchi, mushrooms, seasonings, dressings and eggs, and lower intake of cakes or snacks, alcoholic drinks, pork, rice rolls, ice cream, beverages, hamburgers or pizza, noodles, refined grains, and coffee. The Western dietary pattern was characterized by higher intake of salad, seasonings, dressings, mixed rice, pancakes, eggs, processed seafood, chicken or duck meat, and beef and lower intake of fruits, nuts and seaweed.

**Table 1:** Rotated factor loadings^a^ for dietary patterns identified by factor analysis in the breast cancer survivors

Food or food group	Pattern 1	Pattern 2
Vegetables	0.6432	.
Whole grains	0.6174	.
Soy	0.4563	.
Potatoes	0.3642	.
Fish	0.3637	.
Fruits	0.3302	−0.3071
Yogurt	0.3151	.
Kimchi	0.3120	.
Mushrooms	0.2807	.
Milk	.	.
Other seafood	.	.
Salted seafood	.	.
Rice soup	.	.
Rice cake	.	.
Cakes/snacks	−0.2021	.
Alcoholic drinks	−0.2114	.
Pork	−0.2318	.
Rice rolls	−0.2371	.
Ice cream	−0.2506	.
Beverages	−0.2962	.
Hamburgers/pizza	−0.3035	.
Noodles	−0.3059	.
Refined grains	−0.3761	.
Coffee	−0.4113	.
Salad	.	0.6423
Seasonings	0.2921	0.5166
Dressings	0.2023	0.4742
Mixed rice	.	0.4170
Pancakes	.	0.3199
Eggs	0.2121	0.3016
Processed seafood	.	0.2757
Chicken/duck meat	.	0.2382
Beef	.	0.2236
Vegetable oil	.	.
Tea	.	.
Chocolate/sugar	.	.
Vegetable or fruit juice	.	.
Nuts	.	−0.2004
Seaweed	.	−0.2354

^a^Absolute values greater than 0.2 were presented

### Characteristics of breast cancer survivors according to their dietary patterns

We compared the general characteristics of the participants according to the quartiles of the factor scores for each dietary pattern (Table 2). Breast cancer survivors with higher Healthy dietary pattern scores were older and had a lower proportion of college education or above, compared to breast cancer survivors with lower Healthy dietary pattern scores. In addition, higher Healthy dietary pattern scores were associated with higher levels of physical activity and energy intake. Meanwhile, breast cancer survivors with higher Western dietary pattern scores were younger and had a higher proportion of college education or above, and they had lower levels of physical activity but higher levels of total energy intake, compared to breast cancer survivors with lower Western dietary pattern scores.

**Table 2:** Characteristics of participants according to quartiles of the Healthy and Western dietary pattern

	Quartiles of Healthy dietary pattern				Quartiles of Western dietary pattern			
Variables	Quartile 1 (*n* = 58)	Quartile 2 (*n* = 58)	Quartile 3 (*n* = 58)	Quartile 4 (*n* = 58)	Quartile 1 (*n* = 58)	Quartile 2 (*n* = 58)	Quartile 3 (*n* = 58)	Quartile 4 (*n* = 58)
Age at diagnosis (years), mean(SD)	44.98 (7.51)	47.22 (8.07)	50.21 (8.15)	51.09 (8.42)	49.71 (7.42)	48.86 (7.34)	49.38 (8.64)	45.55 (9.38)
Body mass index at diagnosis (kg/m^2^), mean(SD)	23.23 (3.10)	23.21 (2.60)	23.42 (2.63)	22.72 (2.95)	22.82 (2.47)	23.16 (2.64)	23.18 (2.88)	23.41 (3.27)
Time since surgery (month), mean(SD)	42.66 (37.26)	32.48 (26.67)	36.02 (31.13)	28.25 (23.17)	35.43 (27.84)	34.30 (28.94)	34.30 (30.79)	35.38 (34.02)
Education level^a^, n(%)								
Elementary school	1 (1.75)	1 (1.75)	5 (8.62)	1 (1.79)	5 (8.77)	2 (3.57)	1 (1.72)	0 (0.00)
Middle school	1 (1.75)	2 (3.51)	0 (0.00)	7 (12.50)	4 (7.02)	2 (3.57)	0 (0.00)	4 (7.02)
High school	24 (42.11)	24 (42.11)	31 (53.45)	28 (50.00)	23 (40.35)	28 (50.00)	32 (55.17)	24 (42.11)
College or above	31 (54.39)	30 (52.63)	22 (37.93)	20 (35.71)	25 (43.86)	24 (42.86)	25 (43.10)	29 (50.88)
Marital status, n(%)								
Married or cohabitation	49 (85.96)	46 (80.70)	51 (87.93)	46 (80.70)	49 (87.50)	48 (82.76)	50 (86.21)	45 (78.95)
Unmarried, divorced, or Widowed	8 (14.04)	11 (19.30)	7 (12.07)	11 (19.30)	7 (12.50)	10 (17.24)	8 (13.79)	12 (21.05)
Physical activity (MET-hours/week), mean(SD)	26.52 (29.64)	37.38 (36.61)	40.64 (43.39)	40.47 (28.43)	40.41 (39.80)	40.72 (40.69)	27.77 (21.80)	36.10 (34.93)
Menopausal status^a^, n(%)								
Pre-menopause	26 (44.83)	28 (48.28)	19 (32.76)	11 (18.97)	17 (29.31)	19 (32.76)	23 (39.66)	25 (43.10)
Post-menopause	32 (55.17)	30 (51.72)	39 (67.24)	47 (81.03)	41 (70.69)	39 (67.24)	35 (60.34)	33 (56.90)
Smoking status^a^, n(%)								
Never	49 (92.45)	50 (98.04)	54 (98.18)	49 (96.08)	46 (97.87)	51 (100.00)	54 (94.74)	51 (92.73)
Ever	4 (7.55)	1 (1.96)	1 (1.82)	2 (3.92)	1 (2.13)	0 (0.00)	3 (5.26)	4 (7.27)
Alcohol intake^a^, n(%)								
Never	23 (40.35)	32 (56.14)	30 (51.72)	28 (49.12)	31 (55.36)	33 (56.90)	29 (50.00)	20 (35.09)
Past	20 (35.09)	18 (31.58)	25 (43.10)	25 (43.86)	21 (37.50)	20 (34.48)	22 (37.93)	25 (43.86)
Current	14 (24.56)	7 (12.28)	3 (5.17)	4 (7.02)	4 (7.14)	5 (8.62)	7 (12.07)	12 (21.05)
AJCC stage at diagnosis, n(%)								
I	27 (46.55)	24 (41.38)	29 (50.00)	23 (39.66)	25 (43.10)	25 (43.10)	25 (43.10)	28 (48.28)
II	24 (41.38)	27 (46.55)	18 (31.03)	30 (51.72)	25 (43.10)	26 (44.83)	24 (41.38)	24 (41.38)
III	7 (12.07)	7 (12.07)	11 (18.97)	5 (8.62)	8 (13.79)	7 (12.07)	9 (15.52)	6 (10.34)
Energy intake (kcal/day), mean(SD)	1659.13 (358.84)	1679.76 (347.61)	1719.96 (372.85)	2049.69 (376.88)	1735.57 (351.47)	1648.52 (353.61)	1796.81 (412.43)	1927.63 (415.55)
Dietary supplement use^a,b^, n(%)								
No	15 (25.86)	18 (32.14)	19 (32.76)	17 (30.36)	14 (24.14)	19 (33.33)	20 (35.09)	16 (28.57)
Yes	43 (74.14)	38 (67.86)	39 (67.24)	39 (69.64)	44 (75.86)	38 (66.67)	37 (64.91)	40 (71.43)

*SD* standard deviation, *AJCC* American joint committee on cancer, *MET* metabolic equivalent of task

Median months since surgery (25th, 75th percentiles) was 23.77 (15.22, 46.27) months

^a^Missing value are not shown

^b^Both nutrient supplement and health functional food were considered

### Health-related quality of life according to dietary patterns among breast cancer survivors

Table 3 shows the associations between the Healthy dietary pattern and HRQoL levels in the multivariable models. Breast cancer survivors who had higher Healthy dietary pattern scores tended to have lower dyspnea scores but higher insomnia scores, compared to breast cancer survivors with lower Healthy dietary pattern scores. For dyspnea, the LS mean (95% CI) was 8.86 (5.05-15.52) in the bottom quartile and 2.87 (1.62-5.08) in the top quartile (*p* for trend = 0.005). For insomnia, the LS mean (95% CI) was 11.46 (6.59-19.95) in the bottom quartile and 29.77 (16.87-52.51) in the top quartile (*p* for trend = 0.005). When we adjusted for multiple comparisons, statistically significant associations remained at an FDR of 0.1 (FDR = 0.055 for both dyspnea and insomnia). When we compared the top quartile with the bottom quartile for the Healthy dietary pattern scores, we also found statistically significant differences for dyspnea and insomnia (*p*-contrast = 0.003 for dyspnea and *p*-contrast = 0.01 for insomnia).

**Table 3:** Least squares (LS) means scores^a^ (95% Confidence intervals, CIs) of HRQoL according to quartiles of Healthy dietary pattern in breast cancer survivors

		Quartiles of the Healthy dietary pattern				
Variables	*N* (=232)	Quartile 1	Quartile 2	Quartile 3	Quartile 4	*P* for trend
EORTC QLQ - C30						
Global health status/QoL	220	40.41 (28.37 - 57.55)	37.47 (27.10 - 51.81)	39.93 (28.92 - 55.14)	30.00 (20.83 - 43.21)	0.31
Functioning						
Physical Functioning	228	76.21 (67.82 - 85.65)	79.02 (71.09 - 87.85)	77.80 (69.85 - 86.65)	71.45 (63.52 - 80.37)	0.45
Role Functioning	229	62.82 (49.32 - 80.02)	77.50 (61.80- 97.17)	69.47 (55.05 - 87.66)	65.77 (51.12 - 84.62)	0.84
Emotional Functioning	230	76.24 (63.16 - 92.04)	72.09 (60.45 - 85.96)	60.42 (50.51 - 72.26)	71.77 (58.99 - 87.32)	0.31
Cognitive Functioning	230	74.53 (65.00 - 85.44)	78.52 (69.10 - 89.22)	73.09 (64.18 - 83.23)	70.66 (61.28 - 81.47)	0.48
Social Functioning	230	57.8 (45.69 - 73.12)	63.28 (50.80 - 78.84)	60.55 (48.42 - 75.72)	62.67 (49.05 - 80.05)	0.66
Symptom						
Fatigue	229	27.26 (19.48 - 38.15)	20.26 (14.88 - 27.58)	22.61 (16.52 - 30.94)	23.51 (16.68 - 33.13)	0.55
Nausea and vomiting	230	4.00 (2.48 - 6.44)	2.24 (1.44 - 3.49)	3.71 (2.36 - 5.83)	3.17 (1.93 - 5.21)	0.73
Pain	229	9.84 (5.80 - 16.71)	6.13 (3.77 - 9.97)	11.13 (6.79 - 18.24)	10.64 (6.20 - 18.28)	0.53
Dyspnea^b,c^	228	8.86 (5.05 - 15.52)	4.21 (2.52 - 7.03)	4.75 (2.82 - 8.01)	2.87 (1.62 - 5.08)	0.005
Insomnia^b,c^	228	11.46 (6.59 - 19.95)	12.11 (7.28 - 20.15)	20.41 (12.15 - 34.26)	29.77 (16.87 - 52.51)	0.005
Loss of appetite	228	3.00 (1.74 - 5.15)	3.66 (2.23 - 6.02)	2.65 (1.60 - 4.39)	3.32 (1.91 - 5.79)	0.99
Constipation	228	4.25 (2.40 - 7.54)	5.69 (3.36 - 9.63)	7.01 (4.10 - 11.99)	4.18 (2.32 - 7.50)	0.78
Diarrhea	230	3.04 (1.82 - 5.08)	2.47 (1.53 - 3.99)	2.76 (1.70 - 4.51)	1.79 (1.05 - 3.05)	0.18
Financial impact	230	7.34 (4.12 - 13.07)	4.51 (2.63 - 7.74)	9.47 (5.47 - 16.40)	9.61 (5.27 - 17.53)	0.27
EORTC QLQ-BR23						
Functioning						
Body image	229	40.11 (27.32 - 58.88)	33.23 (23.21 - 47.59)	40.56 (28.06 - 58.62)	28.18 (18.89 - 42.03)	0.30
Sexual functioning	216	2.83 (1.62 - 4.92)	3.89 (2.32 - 6.52)	3.93 (2.33 - 6.65)	3.94 (2.20 - 7.08)	0.33
Future perspective	229	26.93 (15.91 - 45.58)	20.85 (12.74 - 34.10)	21.67 (13.08 - 35.90)	15.43 (8.92 - 26.70)	0.14
Symptom						
Systematic therapy side effects	230	23.34 (17.37 - 31.37)	15.01 (11.39 - 19.79)	22.47 (16.96 - 29.76)	25.97 (19.09 - 35.33)	0.39
Breast symptoms	230	12.10 (7.66 - 19.11)	6.96 (4.54 - 10.68)	10.50 (6.80 - 16.22)	12.11 (7.52 - 19.49)	0.84
Arm symptoms	230	20.54 (13.59 - 31.03)	18.25 (12.41 - 26.84)	21.70 (14.65 - 32.12)	31.48 (20.48 - 48.39)	0.13
Upset by hair loss	151	14.15 (6.70 - 29.89)	32.78 (15.61 - 68.82)	23.84 (12.30 - 46.18)	34.49 (16.45 - 72.32)	0.11

*LS means* least squares means, *95% CI* 95% confidence interval, *HRQoL* health-related quality of life, *EORTC QLQ-C30* European Organization for Research and Treatment of Cancer Quality of Life Questionnaire Core 30, *BR23* breast cancer module 23

^a^Adjusted for age at diagnosis (year; continuous), body mass index at diagnosis (kg/m^2^; continuous), energy intake (kcal/d; continuous), marital status (married or cohabitation, others), education level (high school or below, college or above), physical activity (MET-hr/wk.; continuous), breast cancer stage at diagnosis (I,II,III), time since surgery (months; continuous) and menopausal status at diagnosis (premenopausal, postmenopausal status)

^b^P value for comparing top with bottom quintiles < 0.05

^c^False Discovery Rate (FDR) < 0.1

The Western dietary pattern was marginally significantly associated with the components of physical functioning and constipation among breast cancer survivors (Table 4). For physical functioning, the LS mean (95% CI) was 67.65 (60.64-75.46) in the bottom quartile and 77.41 (69.47-86.24) in the top quartile (*p* for trend = 0.06). For constipation, the LS mean (95% CI) was 4.44 (2.56-7.70) in the bottom quartile and 8.09 (4.69-13.94) in the top quartile (*p* for trend = 0.07). However, the multiple comparison test did not show statistical significance at an FDR of 0.1. In the contrasting comparison of the Western dietary pattern, physical functioning and constipation scores were higher in the top quartile than in the bottom quartile, with marginal significance (*p*-contrast = 0.05 for physical functioning and *p*-contrast = 0.08 for constipation).

**Table 4:** Least squares (LS) means scores^a^ (95% Confidence intervals, CIs) of HRQoL according to quartiles of Western dietary pattern in breast cancer survivors

		Quartiles of Western dietary pattern				
Variables	*N* (=232)	Quartile 1	Quartile 2	Quartile 3	Quartile 4	*P* for trend
EORTC QLQ - C30						
Global health status/QoL	200	33.35 (23.82 - 46.70)	36.10 (25.92 - 50.27)	34.29 (24.56 - 47.89)	43.52 (31.43 - 60.25)	0.22
Functioning						
Physical Functioning	228	67.65 (60.64 - 75.46)	77.67 (69.77 - 86.45)	81.89 (73.67 - 91.03)	77.41 (69.47 - 86.24)	0.06
Role Functioning	229	57.02 (45.20 - 71.93)	70.04 (55.73 - 88.02)	79.94 (63.68 - 100.34)	70.20 (55.45 - 88.88)	0.15
Emotional Functioning	230	63.32 (52.75 – 76.00)	70.77 (59.14 - 84.69)	77.86 (65.12 - 93.10)	66.73 (55.55 - 80.15)	0.61
Cognitive Functioning	230	74.49 (65.26 - 85.03)	70.71 (62.09 - 80.53)	78.88 (69.30 - 89.78)	73.62 (64.47 - 84.06)	0.86
Social Functioning	230	62.80 (50.07 - 78.78)	61.88 (49.52 - 77.32)	66.70 (53.43 - 83.26)	53.96 (42.98 - 67.73)	0.34
Symptom						
Fatigue	229	25.62 (18.52 - 35.43)	20.78 (15.17 - 28.46)	22.48 (16.42 - 30.77)	23.91 (17.34 - 32.96)	0.88
Nausea and vomiting	230	4.96 (3.15 - 7.82)	2.53 (1.62 - 3.95)	2.17 (1.39 - 3.39)	3.84 (2.43 - 6.06)	0.46
Pain	229	12.87 (7.72 - 21.45)	8.12 (4.94 - 13.33)	8.19 (4.99 - 13.44)	8.22 (4.95 - 13.63)	0.21
Dyspnea	228	4.23 (2.45 - 7.30)	6.61 (3.89 - 11.22)	3.76 (2.21 - 6.39)	4.50 (2.62 - 7.73)	0.78
Insomnia	228	24.84 (14.51 - 42.52)	11.00 (6.52 - 18.55)	16.76 (9.96 - 28.19)	18.81 (11.04 - 32.05)	0.75
Loss of appetite	228	3.33 (1.98 - 5.61)	3.08 (1.85 - 5.11)	2.64 (1.59 - 4.37)	3.65 (2.18 - 6.13)	0.83
Constipation	228	4.44 (2.56 - 7.70)	4.46 (2.61 - 7.60)	4.67 (2.74 - 7.96)	8.09 (4.69 - 13.94)	0.07
Diarrhea	230	2.46 (1.50 - 4.04)	1.9 (1.17 - 3.10)	3.28 (2.02 - 5.33)	2.47 (1.50 - 4.06)	0.67
Financial impact	230	9.92 (5.65 - 17.43)	6.97 (4.01 - 12.14)	6.45 (3.72 - 11.20)	6.42 (3.65 - 11.3)	0.24
EORTC QLQ-BR23						
Functioning						
Body image	229	38.50 (26.43 - 56.06)	30.60 (21.23 - 44.12)	41.82 (29.06 - 60.19)	31.51 (21.69 - 45.77)	0.62
Sexual functioning	216	4.15 (2.46 – 7.00)	2.23 (1.32 - 3.76)	4.77 (2.81 - 8.08)	4.08 (2.40 - 6.93)	0.55
Future perspective	229	18.36 (10.99 - 30.67)	22.97 (13.94 - 37.85)	28.15 (17.13 - 46.27)	15.63 (9.39 - 26.03)	0.64
Symptom						
Systematic therapy side effects	230	24.90 (18.63 - 33.29)	19.03 (14.31 - 25.32)	18.20 (13.69 - 24.18)	22.45 (16.78 - 30.04)	0.65
Breast symptoms	230	16.03 (10.35 - 24.83)	7.07 (4.60 - 10.87)	8.36 (5.45 - 12.82)	10.97 (7.07 - 17.01)	0.35
Arm symptoms	230	27.23 (18.22 - 40.71)	21.05 (14.18 - 31.25)	20.81 (14.04 - 30.85)	20.65 (13.8 - 30.91)	0.32
Upset by hair loss	151	24.94 (12.52 - 49.66)	29.96 (14.49 - 61.98)	34.18 (16.69 – 70.00)	17.16 (8.42 - 34.97)	0.44

*LS means* least squares means, *95% CI* 95% confidence interval, *HRQoL* health-related quality of life, *EORTC QLQ- C30* European Organization for Research and Treatment of Cancer Quality of Life Questionnaire Core 30, *BR23* breast cancer module 23

^a^Adjusted for age at diagnosis (year; continuous), body mass index at diagnosis (kg/m^2^; continuous), energy intake (kcal/d; continuous), marital status (married or cohabitation, others), education level (high school or below, college or above), physical activity (MET-hr/wk.; continuous), breast cancer stage at diagnosis (I,II,III), time since surgery (months; continuous) and menopausal status at diagnosis (premenopausal, postmenopausal status)

When we examined the associations between the dietary pattern and HRQoL levels according to the stage at diagnosis, among stage I breast cancer survivors, we found that breast cancer survivors with higher Healthy dietary pattern scores tended to have lower global health status/QoL and dyspnea scores, compared to those with lower Healthy dietary pattern scores (*p* for trend = 0.01 for global health status/QoL and *p* for trend = 0.01 for dyspnea) (Additional file 1: Table S1). In addition, upset by hair loss scores were higher in the top quartile, compared to the bottom quartile (*p*-contrast = 0.01). The multiple comparison test showed significant contrast comparing the top with the bottom quartiles for global health status/QoL, dyspnea, and upset by hair loss at an FDR of 0.1. We observed that stage II or III breast cancer survivors with higher Healthy dietary pattern scores tended to have higher insomnia scores, compared to stage II or III breast cancer survivors with lower Healthy dietary pattern scores (*p* for trend = 0.01) (Additional file 1: Table S2).

Higher Western dietary pattern scores were associated with higher constipation scores among stage I breast cancer survivors (Additional file 1: Table S3). The LS mean (95% CI) was 4.72 (2.09-10.68) in the bottom quartile and 18.85 (8.86-40.10) in the top quartile (*p* for trend = 0.02). However, the Western dietary pattern was not associated with HRQoL levels among stage II or stage III breast cancer survivors (Additional file 1: Table S4).

We examined whether the association between dietary pattern and HRQoL levels varied by menopausal status. We did not observe any significant associations with the Healthy dietary pattern among premenopausal breast cancer survivors (Additional file 1: Table S5). However, among postmenopausal breast cancer survivors, higher Healthy dietary pattern scores were associated with decreasing levels of dyspnea (*p* for trend = 0.003) or diarrhea (*p* for trend = 0.06) and increasing levels of insomnia (*p* for trend = 0.02) (Additional file 1: Table S6). Dyspnea remained significant even after adjusting for multiple comparisons (FDR *P* value = 0.066). The Western dietary pattern was associated with role functioning among premenopausal breast cancer survivors (*p* for trend = 0.06) (Additional file 1: Table S7) and nausea and vomiting among postmenopausal breast cancer survivors (*p* for trend = 0.03, Additional file 1: Table S8).

When we examined the associations according to time since surgery, we observed that breast cancer survivors who had higher Healthy dietary pattern scores tended to have higher upset by hair loss scores, compared to breast cancer survivors with lower Healthy dietary pattern scores who were within 2 years of surgery (*p* for trend = 0.02 for upset by hair loss) (Additional file 1: Table S9). Among breast cancer survivors for whom it had been more than 2 years since their surgery, higher Healthy dietary pattern scores were associated with decreasing levels of dyspnea (*p* for trend = 0.03) but increasing levels of insomnia (*p* for trend = 0.03) and financial impact (*p* for trend = 0.01, Additional file 1: Table S10). We did not observe any statistically significant associations between the Western dietary pattern and HRQoL levels among breast cancer survivors whose surgery was less than 2 years ago and more than 2 years ago (Additional file 1: Tables S11 and S12).

## Discussion

In our cross-sectional study among breast cancer survivors, we identified two dietary patterns: the “Healthy dietary pattern” and the “Western dietary pattern”. We observed that high Healthy dietary pattern scores were significantly associated with decreasing dyspnea scores and increasing insomnia scores. When we limited analysis to stage I or stage II/III survivors, we observed that dyspnea scores decreased only among stage I survivors, but insomnia scores increased only among stage II/III survivors with increasing levels of the Healthy dietary pattern. Along with this observation, upset by hair loss scores increased according to the level of the Healthy dietary pattern among breast cancer survivors who had surgery less than 2 years ago. We also found that stage I breast cancer survivors who had high Western dietary pattern scores had higher constipation scores, compared to stage I breast cancer survivors with low Western dietary pattern scores.

The observation that scores for insomnia or upset by hair loss increased with the Healthy dietary pattern was unexpected. Given the cross-sectional design of this study, the association between dietary factors and distress may be bidirectional. The reason we observed the positive association between Healthy dietary pattern and insomnia or upset by hair loss is not known and warrants further prospective studies.

We found that, among stage I survivors, dyspnea symptom scores were lower among those who followed a Healthy dietary pattern, compared to those with low scores of a Healthy dietary pattern. The potential link between foods, such as fruits and vegetables, whole grains, and fish, characterizing a Healthy dietary pattern, and physiological [19, 20] and psychological well-being [21, 22] may explain the relief of dyspnea by healthy diet. Our findings could be due to chance but warrant further prospective investigation.

For the Western dietary pattern, we found that Korean stage I breast cancer survivors with the Western dietary pattern tended to have constipation symptoms. The low fiber content in the Western diet may partly explain our findings [23]. Similar results were observed in a Japanese study. A cross-sectional study of 3370 Japanese women aged 18 to 20 years showed that women who had a traditional Japanese dietary pattern, characterized by a high intake of rice, miso soup, and soy products, and a low intake of bread and confectionaries, had a significantly lower prevalence of functional constipation [24]. Future studies should confirm this association and determine whether the Western dietary pattern can increase constipation symptoms.

Several cross-sectional studies have suggested a potential link between high diet quality and improved quality of life among cancer survivors. The Iowa Women’s Health Study examined the association between adherence to the World Cancer Research Fund (WCRF) and the American Institute for Cancer Research (AICR) diet and physical activity guidelines and HRQoL among a total of 2193 female cancer survivors who completed the 2004 followup questionnaire of dietary and HRQoL assessments after their cancer diagnosis. In that study, higher adherence to the WCRF/AICR diet and physical activity guidelines was significantly associated with better physical and mental component scores of HRQoL [25].

Several observational and intervention studies also found that adherence to a healthy diet was associated with better scores of quality of life among breast cancer survivors. A US study of 117 female breast cancer survivors observed an inverse association between HEI scores and self-administered depression scores [26]. In the HEAL Study, using the Diet Quality Index and SF-36 scale, breast cancer survivors with good diet quality had higher overall mental health functioning and physical functioning scores than did those with poor diet quality [11]. The same HEAL study examined the association of overall diet quality using the HEI-2010. They found that survivors with better diet quality had lower fatigue levels compared to survivors with lower diet quality [7]. A randomized clinical trial of 735 older long-term survivors of breast, prostate, and colorectal cancer showed that higher diet quality was associated with better physical quality of life among all survivors, including breast cancer [27]. The HEAL study assessed diet more than 2 years after diagnosis; therefore, patients might have completed their primary treatment. The stronger impact on quality of life of behavioral interventions after active treatment compared to interventions during treatment has been emphasized [28]. Breast cancer survivors in our study also completed radiotherapy or chemotherapy treatment, and therefore, their lifestyles may be of great importance to their quality of life. Although we did not assess this association in a prospective way, our findings may emphasize the need of further prospective and interventional studies on dietary modification and improvement of quality of life among long-term breast cancer survivors.

To our knowledge, the present study is the first to investigate an association between the empirically driven dietary patterns and quality of life among breast cancer survivors in Korea. We obtained dietary information using a three-day dietary record along with a food photograph booklet. This may have helped to improve the accuracy of dietary measurements. We were able to use high-quality clinical information collected from a well-established medical records electronic system. Nevertheless, our study has several limitations. We assessed dietary information using a three-day dietary record because there was no food frequency questionnaire available specifically for Korean breast cancer survivors. Although three-day dietary records are often regarded as a gold standard, they may not represent habitual dietary intake. Our study may need to be replicated in a study of other Korean cancer survivor populations where long-term dietary intake after cancer diagnosis is recorded. Given that this was a cross-sectional study with a relatively small sample size, we were unable to determine a causal relationship between dietary pattern and HRQoL levels or to rule out reverse associations. For example, breast cancer survivors with dyspnea could have low appetite, resulting in an inverse association between healthy dietary patterns and dyspnea scores. Quality of life could play a role in eating habits. In addition, we were unable to obtain accurate information on comorbidity at enrollment, which might be associated with quality of life. Classification of menopausal status at diagnosis from median menopausal age could lead to misclassification to some extent; however, only a few had missing information regarding menopausal status. Additionally, the presence of unmeasured or residual confounding factors could not be ruled out. Our results may not be generalizable to all Korean breast cancer survivors.

## Conclusions

We observed that the Healthy dietary pattern was inversely associated with dyspnea but was positively associated with insomnia. The association with dyspnea was mainly derived by the association among patients with stage I breast cancer at diagnosis, and the association for insomnia was more apparent among stage II or III breast cancer survivors than among stage I survivors. In addition, stage I breast cancer survivors who had high Western dietary pattern scores had higher levels of constipation symptoms, compared to stage I breast cancer survivors with low Western dietary pattern scores. Although we cannot rule out the possibility of chance findings, it is important to expand our study to examine how diet plays a role in the improvement of quality of life and survival in breast cancer survivors.

## Additional file

Additional file 1: **Tables S1-S12.** Subgroup analyses. (DOCX 106 kb)

## Abbreviations

ACS: American Cancer Society
AICR: American Institute for Cancer Research
BMI: Body mass index
CAN-Pro: Computer-Aided Nutritional Analysis Program
CI: Confidence interval
EORTC: European Organization for Research and Treatment of Cancer
FDR: False Discovery Rate
GLM: Generalized linear model
HEAL: Health, Eating, Activity, and Lifestyle
HEI: Healthy Eating Index
HRQoL: Health-related quality of life
LS: Least squares
MET: Metabolic equivalent task
QLQ-C30: Quality of Life Questionnaire Core 30
QLQ-BR23: Quality of Life Questionnaire Breast Cancer Module 23
WCRF: World Cancer Research Fund
