# Supplementary material for: Dietary pattern and health-related quality of life among breast cancer survivors
Source: BMC Womens Health. 2018 May 10;18:65. doi: 10.1186/s12905-018-0555-7 (PMC5946513; doi:10.1186/s12905-018-0555-7)
Supplement: Supplementary file 1 — Tables S1-S12. Subgroup analyses. (DOCX 106 kb) [file 12905_2018_555_MOESM1_ESM.docx]

**Table S1. Least squares (LS) means scores^a^ (95% Confidence intervals, CIs) of HRQoL according to quartiles of Healthy dietary pattern in breast cancer survivors, AJCC stage I at diagnosis**

|  |  | **Quartiles of the Healthy dietary pattern** | | | |  |
| --- | --- | --- | --- | --- | --- | --- |
| **Variables** | **N (=103)** | **Quartile 1** | **Quartile 2** | **Quartile 3** | **Quartile 4** | ***P* for trend** |
| **EORTC QLQ-C30** |  |  |  |  |  |  |
| **Global health status/QoL^b,c^** | 92 | 56.29 (35.85 - 88.40) | 51.82 (32.62 - 82.31) | 41.86 (28.16 - 62.22) | 19.92 (11.96 - 33.19) | 0.01 |
| **Functioning** |  |  |  |  |  |  |
| Physical Functioning | 102 | 78.92 (72.03 - 86.47) | 75.71 (69.08 - 82.98) | 78.53 (72.41 - 85.16) | 80.34 (72.34 - 89.21) | 0.77 |
| Role Functioning | 102 | 75.76 (58.85 - 97.52) | 64.93 (50.40 - 83.65) | 74.72 (59.72 - 93.49) | 73.80 (55.25 - 98.58) | 0.96 |
| Emotional Functioning | 102 | 79.39 (54.97 - 114.67) | 67.43 (46.63 - 97.51) | 50.82 (36.67 - 70.44) | 73.00 (47.89 - 111.29) | 0.29 |
| Cognitive Functioning | 102 | 75.34 (64.91 - 87.46) | 69.51 (59.85 - 80.73) | 68.31 (59.84 - 77.97) | 76.72 (64.66 - 91.03) | 0.81 |
| Social Functioning | 102 | 60.83 (43.47 - 85.13) | 74.38 (53.09 - 104.21) | 55.91 (41.49 - 75.34) | 59.10 (40.19 - 86.89) | 0.63 |
| **Symptom** |  |  |  |  |  |  |
| Fatigue | 102 | 31.55 (21.10 - 47.18) | 35.50 (23.70 - 53.15) | 26.78 (18.73 - 38.27) | 32.78 (20.66 - 52.00) | 0.75 |
| Nausea and vomiting | 102 | 4.20 (2.04 - 8.64) | 3.43 (1.67 - 7.08) | 4.46 (2.35 - 8.45) | 3.74 (1.64 - 8.55) | 0.99 |
| Pain | 102 | 10.83 (5.21 - 22.52) | 7.38 (3.54 - 15.37) | 12.41 (6.48 - 23.75) | 8.91 (3.85 - 20.63) | 0.96 |
| Dyspnea^b,c^ | 101 | 10.09 (4.48 - 22.69) | 4.21 (1.88 - 9.42) | 4.00 (1.97 - 8.14) | 1.87 (0.75 - 4.69) | 0.01 |
| Insomnia | 102 | 21.04 (10.12 - 43.72) | 10.78 (5.18 - 22.46) | 18.03 (9.42 - 34.50) | 50.76 (21.94 - 117.44) | 0.20 |
| Loss of appetite | 102 | 1.80 (0.82 - 3.95) | 3.34 (1.52 - 7.36) | 2.02 (1.01 - 4.07) | 2.86 (1.16 - 7.06) | 0.64 |
| Constipation | 102 | 5.22 (2.22 - 12.27) | 10.21 (4.33 - 24.08) | 8.84 (4.14 - 18.89) | 5.11 (1.92 - 13.63) | 0.82 |
| Diarrhea | 102 | 3.08 (1.47 - 6.42) | 2.53 (1.21 - 5.30) | 4.05 (2.11 - 7.78) | 2.05 (0.88 - 4.77) | 0.87 |
| Financial impact | 102 | 4.68 (2.04 - 10.69) | 2.80 (1.22 - 6.41) | 8.16 (3.91 - 16.99) | 9.89 (3.83 - 25.54) | 0.11 |
| **EORTC QLQ-BR23** |  |  |  |  |  |  |
| **Functioning** |  |  |  |  |  |  |
| Body image | 102 | 50.35 (30.60 - 82.83) | 35.62 (21.62 - 58.70) | 55.13 (35.44 - 85.77) | 37.12 (20.97 - 65.70) | 0.80 |
| Sexual functioning | 97 | 2.63 (1.18 - 5.87) | 4.70 (2.05 - 10.75) | 4.28 (2.11 - 8.71) | 4.86 (1.86 - 12.68) | 0.29 |
| Future perspective | 102 | 30.82 (13.99 - 67.87) | 16.75 (7.58 - 36.98) | 18.42 (9.14 - 37.12) | 16.88 (6.83 - 41.75) | 0.29 |
| **Symptom** |  |  |  |  |  |  |
| Systematic therapy side  effects | 102 | 23.82 (15.52 - 36.55) | 16.23 (10.56 - 24.94) | 21.37 (14.61 - 31.25) | 28.99 (17.74 - 47.37) | 0.59 |
| Breast symptoms | 102 | 8.97 (4.94 - 16.28) | 7.88 (4.33 - 14.33) | 8.94 (5.27 - 15.18) | 11.12 (5.61 - 22.04) | 0.67 |
| Arm symptoms | 102 | 16.95 (9.87 - 29.12) | 20.62 (11.98 - 35.50) | 17.77 (10.99 - 28.73) | 18.68 (10.04 - 34.75) | 0.89 |
| Upset by hair loss^b,c^ | 69 | 10.61 (3.55 - 31.72) | 32.98 (10.58 - 102.79) | 13.27 (5.39 - 32.71) | 82.56 (26.24 - 259.77) | 0.08 |

Abbreviations: LS means, least squares means; 95% CI, 95% confidence interval; HRQoL, health-related quality of life; EORTC QLQ-C30, European Organization for Research and Treatment of Cancer Quality of Life Questionnaire Core 30; BR23, breast cancer module 23; AJCC, American Joint Committee on Cancer

^a^ Adjusted for age at diagnosis (year; continuous), body mass index at diagnosis (kg/m^2^; continuous), energy intake (kcal/d; continuous), marital status (married or cohabitation, others), education level (high school or below, college or above), physical activity (MET-hr/wk; continuous), time since surgery (months; continuous) and menopausal status at diagnosis (premenopausal, postmenopausal status)

^b^ P value for comparing top with bottom quintiles <0.05

^c^ False Discovery Rate (FDR) <0.1

**Table S2. Least squares (LS) means scores^a^ (95% Confidence intervals, CIs) of HRQoL according to quartiles of Healthy dietary pattern in breast cancer survivors, AJCC stage II or III at diagnosis**

|  |  | **Quartiles of the Healthy dietary pattern** | | | |  |
| --- | --- | --- | --- | --- | --- | --- |
| **Variables** | **N (=129)** | **Quartile 1** | **Quartile 2** | **Quartile 3** | **Quartile 4** | ***P* for trend** |
| **EORTC QLQ-C30** |  |  |  |  |  |  |
| **Global health status/QoL** | 108 | 31.84 (19.13 – 53.00) | 29.78 (19.50 - 45.47) | 36.82 (22.80 - 59.44) | 33.24 (20.29 - 54.45) | 0.77 |
| **Functioning** |  |  |  |  |  |  |
| Physical Functioning | 126 | 70.54 (57.96 - 85.86) | 81.80 (69.41 - 96.40) | 78.13 (64.81 - 94.19) | 64.50 (54.06 - 76.96) | 0.41 |
| Role Functioning | 127 | 46.36 (32.01 - 67.15) | 86.18 (62.11 - 119.59) | 62.99 (43.11 - 92.04) | 53.94 (37.80 - 76.96) | 0.77 |
| Emotional Functioning | 128 | 66.75 (58.12 - 76.66) | 74.23 (65.68 - 83.88) | 69.41 (60.37 - 79.80) | 65.15 (57.08 - 74.35) | 0.67 |
| Cognitive Functioning | 128 | 70.47 (57.10 - 86.99) | 83.41 (69.26 - 100.45) | 75.86 (61.36 - 93.78) | 64.02 (52.37 - 78.26) | 0.41 |
| Social Functioning | 128 | 52.77 (38.46 - 72.41) | 57.81 (43.72 - 76.44) | 64.12 (46.62 - 88.19) | 63.00 (46.59 - 85.20) | 0.35 |
| **Symptom** |  |  |  |  |  |  |
| Fatigue | 127 | 31.45 (19.10- 51.77) | 16.47 (10.77 - 25.20) | 23.69 (14.60 - 38.44) | 27.59 (17.45 - 43.64) | 0.93 |
| Nausea and vomiting | 128 | 4.34 (2.35 - 8.01) | 1.63 (0.95 - 2.80) | 3.33 (1.80 - 6.18) | 3.05 (1.70 - 5.48) | 0.68 |
| Pain | 127 | 11.28 (5.42 - 23.49) | 5.65 (3.02 - 10.56) | 11.09 (5.44 - 22.61) | 14.18 (7.22 - 27.84) | 0.41 |
| Dyspnea | 127 | 9.10 (4.23 - 19.58) | 4.44 (2.31 - 8.54) | 5.76 (2.74 - 12.13) | 4.02 (1.99 - 8.13) | 0.15 |
| Insomnia^b^ | 126 | 8.52 (3.97 - 18.30) | 13.85 (7.21 - 26.60) | 27.19 (12.92 - 57.22) | 26.13 (12.86 - 53.09) | 0.01 |
| Loss of appetite | 126 | 4.45 (2.21 - 8.95) | 3.09 (1.70 - 5.61) | 3.12 (1.58 - 6.16) | 3.58 (1.87 - 6.84) | 0.65 |
| Constipation | 126 | 5.32 (2.51 - 11.27) | 4.91 (2.59 - 9.32) | 7.56 (3.62 - 15.79) | 4.55 (2.28 - 9.07) | 0.91 |
| Diarrhea | 128 | 3.22 (1.61 - 6.42) | 2.52 (1.37 - 4.64) | 1.91 (0.96 - 3.84) | 1.63 (0.84 - 3.14) | 0.11 |
| Financial impact | 128 | 13.15 (6.15 - 28.13) | 5.95 (3.04 - 11.64) | 10.74 (4.99 - 23.10) | 12.44 (6.02 - 25.70) | 0.87 |
| **EORTC QLQ-BR23** |  |  |  |  |  |  |
| **Functioning** |  |  |  |  |  |  |
| Body image | 127 | 39.52 (23.14 - 67.48) | 32.53 (20.27 - 52.20) | 33.96 (19.69 - 58.59) | 24.96 (14.97 - 41.59) | 0.22 |
| Sexual functioning | 119 | 2.73 (1.30 - 5.73) | 3.94 (2.05 - 7.56) | 3.78 (1.80 - 7.95) | 3.26 (1.61 - 6.61) | 0.72 |
| Future perspective | 127 | 21.71 (11.03 - 42.71) | 23.74 (13.05 - 43.17) | 25.01 (12.55 - 49.85) | 12.23 (6.41 - 23.33) | 0.23 |
| **Symptom** |  |  |  |  |  |  |
| Systematic therapy side  effects | 128 | 25.05 (16.97 - 36.99) | 15.08 (10.69 - 21.27) | 24.95 (16.85 - 36.95) | 28.68 (19.77 - 41.59) | 0.35 |
| Breast symptoms | 128 | 16.91 (9.05 - 31.59) | 6.16 (3.55 - 10.71) | 12.27 (6.53 - 23.04) | 15.42 (8.49 - 27.99) | 0.86 |
| Arm symptoms | 128 | 24.28 (13.74 - 42.89) | 14.68 (8.88 - 24.27) | 25.25 (14.23 - 44.79) | 43.64 (25.36 - 75.10) | 0.07 |
| Upset by hair loss | 82 | 18.15 (7.01 - 46.97) | 36.16 (14.46 - 90.42) | 36.75 (14.55 - 92.80) | 21.27 (8.39 - 53.92) | 0.71 |

Abbreviations: LS means, least squares means; 95% CI, 95% confidence interval; HRQoL, health-related quality of life; EORTC QLQ-C30, European Organization for Research and Treatment of Cancer Quality of Life Questionnaire Core 30; BR23, breast cancer module 23; AJCC, American Joint Committee on Cancer

^a^ Adjusted for age at diagnosis (year; continuous), body mass index at diagnosis (kg/m^2^; continuous), energy intake (kcal/d; continuous), marital status (married or cohabitation, others), education level (high school or below, college or above), physical activity (MET-hr/wk; continuous), time since surgery (months; continuous) and menopausal status at diagnosis (premenopausal, postmenopausal status)

^b^ P value for comparing top with bottom quintiles <0.05

**Table S3. Least squares (LS) means scores^a^ (95% Confidence intervals, CIs) of HRQoL according to quartiles of Western dietary pattern in breast cancer survivors, AJCC stage I at diagnosis**

|  |  | **Quartiles of Western dietary pattern** | | | |  |
| --- | --- | --- | --- | --- | --- | --- |
| **Variables** | **N (=103)** | **Quartile 1** | **Quartile 2** | **Quartile 3** | **Quartile 4** | ***P* for trend** |
| **EORTC QLQ-C30** |  |  |  |  |  |  |
| **Global health status/QoL** | 92 | 30.37 (19.09 - 48.31) | 41.63 (26.33 - 65.80) | 39.21 (24.98 - 61.53) | 40.61 (26.33 - 62.64) | 0.46 |
| **Functioning** |  |  |  |  |  |  |
| Physical Functioning | 102 | 73.38 (67.01 - 80.36) | 75.33 (69.03 - 82.21) | 81.46 (74.65 - 88.91) | 78.89 (72.53 - 85.80) | 0.18 |
| Role Functioning | 102 | 62.17 (48.23 - 80.13) | 67.63 (52.97 - 86.35) | 73.91 (57.89 - 94.37) | 75.84 (59.97 - 95.91) | 0.24 |
| Emotional Functioning | 102 | 57.11 (39.07 - 83.49) | 57.63 (39.99 - 83.06) | 75.05 (52.07 - 108.16) | 60.29 (42.43 - 85.67) | 0.80 |
| Cognitive Functioning | 102 | 73.20 (63.09 - 84.93) | 65.88 (57.10 - 76.01) | 76.49 (66.30 - 88.26) | 70.71 (61.63 - 81.13) | 0.98 |
| Social Functioning | 102 | 64.91 (46.31 - 90.98) | 62.20 (44.95 - 86.09) | 61.33 (44.31 - 84.89) | 59.01 (43.18 - 80.64) | 0.64 |
| **Symptom** |  |  |  |  |  |  |
| Fatigue | 102 | 37.77 (25.30 - 56.39) | 36.27 (24.66 - 53.35) | 27.82 (18.91 - 40.92) | 28.99 (20.01 - 42.01) | 0.24 |
| Nausea and vomiting | 102 | 8.03 (3.98 - 16.23) | 3.66 (1.86 - 7.20) | 2.48 (1.26 - 4.88) | 5.46 (2.85 - 10.47) | 0.51 |
| Pain | 102 | 14.89 (7.13 - 31.08) | 11.04 (5.44 - 22.42) | 10.94 (5.39 - 22.22) | 8.25 (4.18 - 16.30) | 0.28 |
| Dyspnea | 101 | 3.23 (1.42 - 7.36) | 5.35 (2.42 - 11.80) | 3.55 (1.59 - 7.91) | 5.11 (2.38 - 10.95) | 0.55 |
| Insomnia | 102 | 26.36 (12.20 - 56.98) | 21.46 (10.22 - 45.06) | 15.39 (7.33 - 32.32) | 29.42 (14.42 - 60.03) | 0.73 |
| Loss of appetite | 102 | 2.16 (0.96 - 4.84) | 3.54 (1.62 - 7.70) | 2.16 (0.99 - 4.71) | 2.81 (1.33 - 5.94) | 0.63 |
| Constipation^b^ | 102 | 4.72 (2.09 - 10.68) | 6.95 (3.17 - 15.23) | 3.67 (1.67 - 8.05) | 18.85 (8.86 - 40.10) | 0.02 |
| Diarrhea | 102 | 3.69 (1.77 - 7.67) | 2.60 (1.29 - 5.26) | 2.17 (1.07 - 4.39) | 3.81 (1.93 - 7.49) | 0.92 |
| Financial impact | 102 | 11.89 (5.11 - 27.70) | 6.28 (2.78 - 14.17) | 3.59 (1.59 - 8.11) | 7.22 (3.30 - 15.78) | 0.41 |
| **EORTC QLQ-BR23** |  |  |  |  |  |  |
| **Functioning** |  |  |  |  |  |  |
| Body image | 102 | 47.22 (28.93 - 77.08) | 32.54 (20.30 - 52.15) | 63.50 (39.62 - 101.77) | 39.90 (25.36 - 62.78) | 0.97 |
| Sexual functioning | 97 | 4.54 (2.07 - 9.95) | 2.27 (1.07 - 4.80) | 9.89 (4.39 - 22.28) | 2.23 (1.06 - 4.70) | 0.35 |
| Future perspective | 102 | 12.95 (5.89 - 28.45) | 14.69 (6.89 - 31.35) | 37.11 (17.39 - 79.18) | 14.74 (7.11 - 30.53) | 0.69 |
| **Symptom** |  |  |  |  |  |  |
| Systematic therapy side  effects | 102 | 26.74 (17.46 - 40.94) | 25.43 (16.88 - 38.32) | 15.75 (10.45 - 23.74) | 24.76 (16.70 - 36.73) | 0.59 |
| Breast symptoms | 102 | 17.10 (9.56 - 30.58) | 9.50 (5.43 - 16.61) | 7.45 (4.26 - 13.04) | 8.27 (4.83 - 14.16) | 0.07 |
| Arm symptoms | 102 | 29.02 (17.01 - 49.51) | 22.60 (13.52 - 37.80) | 13.59 (8.13 - 22.73) | 18.60 (11.35 - 30.48) | 0.17 |
| Upset by hair loss | 69 | 31.01 (11.26 - 85.38) | 45.56 (14.71 - 141.08) | 16.61 (5.38 - 51.30) | 19.83 (7.87 - 49.98) | 0.32 |

Abbreviations: LS means, least squares means; 95% CI, 95% confidence interval; HRQoL, health-related quality of life; EORTC QLQ-C30, European Organization for Research and Treatment of Cancer Quality of Life Questionnaire Core 30; BR23, breast cancer module 23; AJCC, American Joint Committee on Cancer

^a^ Adjusted for age at diagnosis (year; continuous), body mass index at diagnosis (kg/m^2^; continuous), energy intake (kcal/d; continuous), marital status (married or cohabitation, others), education level (high school or below, college or above), physical activity (MET-hr/wk; continuous), time since surgery (months; continuous) and menopausal status at diagnosis (premenopausal, postmenopausal status)

^b^ P value for comparing top with bottom quintiles <0.05

**Table S4. Least squares (LS) means scores^a^ (95% Confidence intervals, CIs) of HRQoL according to quartiles of Western dietary pattern in breast cancer survivors, AJCC stage II or III at diagnosis**

|  |  | **Quartiles of Western dietary pattern** | | | |  |
| --- | --- | --- | --- | --- | --- | --- |
| **Variables** | **N (=129)** | **Quartile 1** | **Quartile 2** | **Quartile 3** | **Quartile 4** | ***P* for trend** |
| **EORTC QLQ-C30** |  |  |  |  |  |  |
| **Global health status/QoL** | 108 | 29.84 (18.66 - 47.72) | 27.32 (17.57 - 42.49) | 33.67 (20.37 - 55.65) | 41.18 (26.08 - 65.02) | 0.23 |
| **Functioning** |  |  |  |  |  |  |
| Physical Functioning | 126 | 62.49 (52.44 - 74.46) | 78.71 (66.37 - 93.34) | 80.26 (67.12 - 95.97) | 75.30 (62.72 - 90.41) | 0.15 |
| Role Functioning | 127 | 49.35 (34.89 - 69.80) | 67.41 (48.04 - 94.60) | 80.18 (55.82 - 115.18) | 58.60 (40.20- 85.42) | 0.45 |
| Emotional Functioning | 128 | 62.76 (55.36 - 71.16) | 75.58 (66.85 - 85.46) | 73.17 (64.17 - 83.43) | 65.57 (57.27 - 75.07) | 0.78 |
| Cognitive Functioning | 128 | 71.54 (58.77 - 87.09) | 71.68 (59.14 - 86.88) | 81.01 (65.96 - 99.49) | 71.95 (58.21 - 88.93) | 0.83 |
| Social Functioning | 128 | 60.59 (45.44 - 80.78) | 60.32 (45.52 - 79.92) | 71.45 (52.89 - 96.51) | 47.27 (34.67 - 64.44) | 0.29 |
| **Symptom** |  |  |  |  |  |  |
| Fatigue | 127 | 25.88 (16.29 - 41.12) | 18.49 (11.91 - 28.70) | 23.53 (14.67 - 37.75) | 27.36 (16.86 - 44.40) | 0.68 |
| Nausea and vomiting | 128 | 4.07 (2.30 – 7.20) | 2.18 (1.25 – 3.82) | 1.96 (1.08 – 3.57) | 3.19 (1.72 – 5.91) | 0.59 |
| Pain | 127 | 13.95 (7.07 - 27.53) | 8.17 (4.29 - 15.58) | 7.76 (3.88 - 15.53) | 9.42 (4.63 - 19.18) | 0.44 |
| Dyspnea | 127 | 5.40 (2.67 - 10.92) | 7.77 (3.98 - 15.16) | 4.00 (1.95 - 8.21) | 4.02 (1.93 - 8.40) | 0.34 |
| Insomnia | 126 | 32.07 (16.00 - 64.27) | 9.05 (4.64 - 17.63) | 22.49 (11.06 - 45.74) | 15.35 (7.40 - 31.83) | 0.34 |
| Loss of appetite | 126 | 4.60 (2.45 - 8.65) | 2.39 (1.31 - 4.38) | 2.80 (1.47 - 5.33) | 4.96 (2.56 - 9.62) | 0.73 |
| Constipation | 126 | 5.39 (2.69 - 10.79) | 4.05 (2.11 - 7.78) | 7.15 (3.54 - 14.41) | 5.74 (2.80 - 11.77) | 0.67 |
| Diarrhea | 128 | 1.96 (1.05 - 3.65) | 1.75 (0.95 - 3.21) | 4.80 (2.50 - 9.19) | 1.86 (0.95 - 3.63) | 0.73 |
| Financial impact | 128 | 10.05 (4.95 - 20.42) | 9.74 (4.87 - 19.48) | 12.26 (5.84 - 25.72) | 7.46 (3.48 - 16.02) | 0.61 |
| **EORTC QLQ-BR23** |  |  |  |  |  |  |
| **Functioning** |  |  |  |  |  |  |
| Body image | 127 | 35.88 (21.72 - 59.25) | 30.87 (18.98 - 50.21) | 31.95 (19.00 - 53.73) | 29.43 (17.24 - 50.24) | 0.60 |
| Sexual functioning | 119 | 3.57 (1.87 - 6.83) | 2.08 (1.07 - 4.04) | 3.02 (1.53 - 5.98) | 6.49 (3.20 - 13.17) | 0.12 |
| Future perspective | 127 | 19.79 (10.52 - 37.23) | 26.91 (14.58 - 49.67) | 20.93 (10.88 - 40.30) | 12.70 (6.47 - 24.90) | 0.24 |
| **Symptom** |  |  |  |  |  |  |
| Systematic therapy side  effects | 128 | 26.75 (18.53 - 38.62) | 17.71 (12.37 - 25.37) | 22.58 (15.39 - 33.15) | 22.85 (15.39 - 33.94) | 0.75 |
| Breast symptoms | 128 | 16.76 (9.40 - 29.87) | 6.41 (3.64 - 11.28) | 10.33 (5.65 - 18.91) | 15.59 (8.36 - 29.05) | 0.84 |
| Arm symptoms | 128 | 25.57 (14.86 – 44.00) | 21.31 (12.53 - 36.23) | 29.88 (16.94 - 52.68) | 22.45 (12.51 - 40.29) | 0.89 |
| Upset by hair loss | 82 | 19.11 (7.86 - 46.49) | 25.02 (10.65 - 58.80) | 60.84 (25.03 - 147.89) | 19.42 (7.26 - 51.90) | 0.66 |

Abbreviations: LS means, least squares means; 95% CI, 95% confidence interval; HRQoL, health-related quality of life; EORTC QLQ-C30, European Organization for Research and Treatment of Cancer Quality of Life Questionnaire Core 30; BR23, breast cancer module 23; AJCC, American Joint Committee on Cancer

^a^ Adjusted for age at diagnosis (year; continuous), body mass index at diagnosis (kg/m^2^; continuous), energy intake (kcal/d; continuous), marital status (married or cohabitation, others), education level (high school or below, college or above), physical activity (MET-hr/wk; continuous), time since surgery (months; continuous) and menopausal status at diagnosis (premenopausal, postmenopausal status)

**Table S5. Least squares (LS) means scores^a^ (95% Confidence intervals, CIs) of HRQoL according to quartiles of Healthy dietary pattern in breast cancer survivors, premenopausal status at diagnosis**

|  |  | **Quartiles of Healthy dietary pattern** | | | |  |
| --- | --- | --- | --- | --- | --- | --- |
| **Variables** | **N (=84)** | **Quartile 1** | **Quartile 2** | **Quartile 3** | **Quartile 4** | ***P* for trend** |
| **EORTC QLQ-C30** |  |  |  |  |  |  |
| **Global health status/QoL** | 74 | 34.51 (20.33 - 58.58) | 33.44 (19.98 - 55.97) | 42.91 (24.01 - 76.70) | 53.23 (24.72 - 114.62) | 0.34 |
| **Functioning** |  |  |  |  |  |  |
| Physical Functioning | 83 | 78.15 (60.12 - 101.59) | 81.36 (64.16 - 103.18) | 79.20 (59.79 - 104.92) | 52.20 (36.59 - 74.48) | 0.19 |
| Role Functioning | 83 | 53.60 (32.50 - 88.38) | 78.62 (49.98 - 123.67) | 56.82 (33.23 - 97.13) | 46.58 (23.65 - 91.74) | 0.76 |
| Emotional Functioning | 83 | 80.40 (59.50 - 108.63) | 77.63 (59.11 - 101.95) | 56.84 (41.17 - 78.49) | 55.82 (37.12 - 83.92) | 0.06 |
| Cognitive Functioning | 83 | 70.19 (59.53 - 82.75) | 78.43 (67.57 - 91.04) | 65.37 (54.79 - 77.99) | 69.74 (55.79 - 87.17) | 0.62 |
| Social Functioning | 83 | 51.79 (32.87 - 81.61) | 55.94 (37.06 - 84.45) | 58.65 (36.02 - 95.50) | 72.73 (39.27 - 134.69) | 0.40 |
| **Symptom** |  |  |  |  |  |  |
| Fatigue | 83 | 28.34 (16.73 - 48.02) | 17.29 (10.73 - 27.88) | 21.54 (12.24 - 37.91) | 28.55 (13.97 - 58.35) | 0.84 |
| Nausea and vomiting | 83 | 5.70 (2.60 - 12.47) | 1.81 (0.89 - 3.67) | 4.24 (1.83 - 9.82) | 2.89 (1.00 - 8.34) | 0.46 |
| Pain | 83 | 7.16 (3.15 - 16.27) | 5.30 (2.52 - 11.16) | 7.38 (3.06 - 17.81) | 9.26 (3.04 - 28.20) | 0.70 |
| Dyspnea | 83 | 4.54 (1.83 - 11.28) | 2.71 (1.19 - 6.18) | 4.24 (1.60 - 11.24) | 2.80 (0.82 - 9.61) | 0.68 |
| Insomnia | 83 | 10.81 (4.47 - 26.15) | 14.79 (6.65 - 32.94) | 12.03 (4.67 - 31.04) | 35.37 (10.68 - 117.15) | 0.23 |
| Loss of appetite | 83 | 2.73 (1.20 - 6.17) | 2.40 (1.14 - 5.03) | 3.22 (1.34 - 7.74) | 2.14 (0.71 - 6.48) | 0.94 |
| Constipation | 83 | 4.04 (1.64 - 9.95) | 5.42 (2.39 - 12.25) | 5.31 (2.02 - 13.97) | 4.81 (1.42 - 16.33) | 0.73 |
| Diarrhea | 83 | 2.03 (0.91 - 4.56) | 2.99 (1.44 - 6.21) | 3.79 (1.59 - 9.03) | 2.62 (0.87 - 7.83) | 0.44 |
| Financial impact | 83 | 5.26 (2.07 - 13.40) | 3.03 (1.30 - 7.06) | 9.69 (3.56 - 26.41) | 9.02 (2.54 - 32.03) | 0.27 |
| **EORTC QLQ-BR23** |  |  |  |  |  |  |
| **Functioning** |  |  |  |  |  |  |
| Body image | 83 | 42.89 (22.86 - 80.45) | 33.19 (18.78 - 58.67) | 55.01 (28.02 - 107.98) | 17.17 (7.32 - 40.29) | 0.38 |
| Sexual functioning | 83 | 2.21 (0.99 - 4.95) | 5.74 (2.76 - 11.92) | 6.00 (2.53 - 14.26) | 2.60 (0.87 - 7.76) | 0.38 |
| Future perspective | 83 | 23.62 (9.93 - 56.17) | 25.70 (11.73 - 56.33) | 24.54 (9.69 - 62.14) | 12.01 (3.71 - 38.85) | 0.50 |
| **Symptom** |  |  |  |  |  |  |
| Systematic therapy side  effects | 83 | 22.39 (14.51 - 34.53) | 14.57 (9.84 - 21.57) | 30.50 (19.16 - 48.54) | 33.29 (18.50 - 59.91) | 0.14 |
| Breast symptoms | 83 | 9.23 (4.69 - 18.17) | 5.45 (2.95 - 10.05) | 7.59 (3.67 - 15.68) | 11.69 (4.67 - 29.25) | 0.81 |
| Arm symptoms | 83 | 14.30 (7.80 - 26.22) | 17.71 (10.23 - 30.67) | 29.98 (15.66 - 57.41) | 26.29 (11.57 - 59.77) | 0.08 |
| Upset by hair loss | 54 | 21.36 (6.24 - 73.09) | 43.99 (13.37 - 144.71) | 25.70 (7.71 - 85.69) | 46.25 (10.22 - 209.34) | 0.50 |

Abbreviations: LS means, least squares means; 95% CI, 95% confidence interval; HRQoL, health-related quality of life; EORTC QLQ-C30, European Organization for Research and Treatment of Cancer Quality of Life Questionnaire Core 30; BR23, breast cancer module 23; AJCC, American Joint Committee on Cancer

^a^ Adjusted for age (year; continuous), body mass index (kg/m^2^; continuous), energy intake (kcal/d; continuous), marital status (married or cohabitation, others), education level (high school or below, college or above), breast cancer stage at diagnosis (I,II,III), physical activity (MET-hr/wk; continuous) and time since surgery (months; continuous)

**Table S6. Least squares (LS) means scores^a^ (95% Confidence intervals, CIs) of HRQoL according to quartiles of Healthy dietary pattern in breast cancer survivors, postmenopausal status at diagnosis**

|  |  | | **Quartiles of Healthy dietary pattern** | | | |  |
| --- | --- | --- | --- | --- | --- | --- | --- |
| **Variables** | **N (=148)** | | **Quartile 1** | **Quartile 2** | **Quartile 3** | **Quartile 4** | ***P* for trend** |
| **EORTC QLQ-C30** |  | |  |  |  |  |  |
| **Global health status/QoL** | 126 | | 39.25 (23.99 - 64.23) | 39.13 (24.92 - 61.46) | 34.66 (23.23 - 51.72) | 24.08 (15.56 - 37.27) | 0.09 |
| **Functioning** |  | |  |  |  |  |  |
| Physical Functioning | 145 | | 72.32 (65.81 - 79.47) | 75.99 (69.61 - 82.95) | 76.48 (70.57 - 82.90) | 75.29 (69.26 - 81.85) | 0.47 |
| Role Functioning | 146 | | 63.32 (49.55 - 80.90) | 73.52 (57.88 - 93.38) | 74.48 (59.65 - 93.02) | 69.94 (55.72 - 87.78) | 0.50 |
| Emotional Functioning | 147 | | 70.86 (55.10 - 91.13) | 67.84 (53.06 - 86.73) | 60.90 (48.65 - 76.23) | 75.53 (59.81 - 95.38) | 0.84 |
| Cognitive Functioning | 147 | | 75.99 (62.14 - 92.94) | 75.28 (61.85 - 91.63) | 73.98 (61.81 - 88.54) | 69.48 (57.65 - 83.75) | 0.46 |
| Social Functioning | 147 | | 58.84 (44.83 - 77.23) | 70.95 (54.41 - 92.53) | 61.85 (48.52 - 78.84) | 60.75 (47.21 - 78.19) | 0.97 |
| **Symptom** |  | |  |  |  |  |  |
| Fatigue | 146 | | 28.46 (18.39 - 44.03) | 22.87 (15.08 - 34.66) | 23.82 (16.29 - 34.85) | 22.59 (15.22 - 33.53) | 0.42 |
| Nausea and vomiting | 147 | | 3.70 (1.99 - 6.90) | 2.83 (1.55 - 5.20) | 3.94 (2.26 - 6.86) | 3.54 (1.99 - 6.30) | 0.91 |
| Pain | 146 | | 16.46 (8.28 - 32.73) | 6.85 (3.56 - 13.18) | 14.20 (7.80 - 25.86) | 13.94 (7.48 - 25.96) | 0.92 |
| Dyspnea^b,c^ | 145 | | 15.53 (7.51 - 32.11) | 6.54 (3.29 - 12.98) | 5.68 (3.04 - 10.64) | 4.10 (2.14 - 7.86) | 0.003 |
| Insomnia | 145 | | 13.72 (6.69 - 28.11) | 9.52 (4.81 - 18.87) | 27.86 (14.90 - 52.06) | 29.14 (15.19 - 55.90) | 0.02 |
| Loss of appetite | 145 | | 3.74 (1.81 - 7.73) | 4.31 (2.16 - 8.62) | 2.44 (1.29 - 4.59) | 3.60 (1.86 - 6.96) | 0.66 |
| Constipation | 145 | | 4.63 (2.16 - 9.94) | 7.05 (3.41 - 14.60) | 10.16 (5.21 - 19.81) | 4.92 (2.46 - 9.83) | 0.74 |
| Diarrhea^b^ | 147 | | 4.10 (2.08 - 8.09) | 2.35 (1.21 - 4.56) | 2.72 (1.48 - 4.98) | 1.72 (0.92 - 3.24) | 0.06 |
| Financial impact | 147 | | 11.93 (5.69 - 24.98) | 5.86 (2.85 - 12.07) | 10.01 (5.17 - 19.36) | 13.07 (6.58 - 25.94) | 0.62 |
| **EORTC QLQ-BR23** |  | |  |  |  |  |  |
| **Functioning** |  | |  |  |  |  |  |
| Body image | 146 | | 38.99 (23.65 - 64.28) | 37.19 (22.82 - 60.59) | 37.39 (23.82 - 58.69) | 34.66 (21.80 - 55.10) | 0.71 |
| Sexual functioning | 133 | | 2.83 (1.29 - 6.21) | 2.63 (1.23 - 5.63) | 2.70 (1.34 - 5.41) | 4.23 (1.98 - 9.02) | 0.40 |
| Future perspective | 146 | | 26.42 (13.26 - 52.63) | 19.13 (9.76 - 37.49) | 19.90 (10.69 - 37.04) | 16.09 (8.49 - 30.49) | 0.27 |
| **Symptom** |  | |  |  |  |  |  |
| Systematic therapy side  effects | 147 | | 25.55 (17.03 - 38.34) | 15.91 (10.71 - 23.65) | 19.99 (13.91 - 28.71) | 24.83 (17.04 - 36.17) | 0.90 |
| Breast symptoms | 147 | | 16.90 (9.16 - 31.20) | 8.03 (4.41 - 14.60) | 11.95 (6.91 - 20.65) | 14.77 (8.36 - 26.07) | 0.95 |
| Arm symptoms | 147 | 34.10 (20.05 – 58.00) | | 18.61 (11.08 - 31.25) | 19.80 (12.32 - 31.80) | 40.29 (24.62 - 65.94) | 0.60 |
| Upset by hair loss | 97 | | 12.43 (4.55 - 33.99) | 29.72 (10.97 - 80.51) | 28.68 (12.47 - 65.99) | 32.78 (13.61 - 78.94) | 0.14 |

Abbreviations: LS means, least squares means; 95% CI, 95% confidence interval; HRQoL, health-related quality of life; EORTC QLQ-C30, European Organization for Research and Treatment of Cancer Quality of Life Questionnaire Core 30; BR23, breast cancer module 23; AJCC, American Joint Committee on Cancer

^a^ Adjusted for age (year; continuous), body mass index (kg/m^2^; continuous), energy intake (kcal/d; continuous), marital status (married or cohabitation, others), education level (high school or below, college or above), breast cancer stage at diagnosis (I,II,III), physical activity (MET-hr/wk; continuous) and time since surgery (months; continuous)

^b^ P value for comparing top with bottom quintiles <0.05

^c^ False Discovery Rate (FDR) <0.1

**Table S7.** **Least squares (LS) means scores^a^ (95% Confidence intervals, CIs) of HRQoL according to quartiles of the Western dietary pattern in breast cancer survivors, premenopausal status at diagnosis**

|  |  | **Quartiles of Western dietary pattern** | | | | |  |
| --- | --- | --- | --- | --- | --- | --- | --- |
| **Variables** | **N (=84)** | **Quartile 1** | **Quartile 2** | **Quartile 3** | | **Quartile 4** | ***P* for trend** |
| **EORTC QLQ-C30** |  |  |  |  | |  |  |
| **Global health status/QoL** | 74 | 26.90 (14.87 - 48.66) | 54.93 (31.91 - 94.55) | 47.12 (28.59 - 77.64) | | 27.73 (16.57 - 46.42) | 0.63 |
| **Functioning** |  |  |  |  | |  |  |
| Physical Functioning | 83 | 57.74 (42.62 - 78.23) | 76.08 (58.41 - 99.08) | 82.55 (63.67 - 107.02) | | 75.97 (58.29 - 99.01) | 0.18 |
| Role Functioning | 83 | 34.15 (19.52 - 59.75) | 57.89 (35.59 - 94.18) | 79.80 (49.47 - 128.74) | | 65.55 (40.24 - 106.77) | 0.06 |
| Emotional Functioning | 83 | 47.50 (33.86 - 66.62) | 73.93 (55.07 - 99.23) | 82.66 (61.90 - 110.40) | | 68.84 (51.25 - 92.48) | 0.13 |
| Cognitive Functioning | 83 | 62.08 (51.96 - 74.18) | 75.14 (64.36 - 87.73) | 84.58 (72.64 - 98.49) | | 62.61 (53.61 - 73.13) | 0.83 |
| Social Functioning | 83 | 59.50 (35.59 - 99.48) | 59.65 (38.15 - 93.27) | 71.84 (46.30 - 111.47) | | 43.05 (27.50 - 67.40) | 0.27 |
| **Symptom** |  |  |  |  | |  |  |
| Fatigue | 83 | 28.56 (15.53 - 52.52) | 17.10 (10.07 - 29.05) | 21.93 (13.03 - 36.92) | | 27.83 (16.36 - 47.34) | 0.72 |
| Nausea and vomiting | 83 | 3.60 (1.47 - 8.79) | 2.61 (1.20 - 5.68) | 1.83 (0.85 - 3.92) | | 7.06 (3.24 - 15.39) | 0.15 |
| Pain | 83 | 8.13 (3.19 - 20.71) | 6.14 (2.72 - 13.86) | 4.60 (2.07 - 10.22) | | 10.06 (4.45 - 22.74) | 0.63 |
| Dyspnea | 83 | 3.07 (1.09 - 8.69) | 5.46 (2.21 - 13.49) | 2.36 (0.97 - 5.75) | | 3.42 (1.38 - 8.47) | 0.83 |
| Insomnia | 83 | 21.75 (7.75 - 61.03) | 12.61 (5.14 - 30.94) | 13.79 (5.71 - 33.31) | | 15.22 (6.19 - 37.44) | 0.68 |
| Loss of appetite | 83 | 2.82 (1.12 - 7.12) | 2.18 (0.97 - 4.88) | 1.90 (0.86 - 4.20) | | 4.14 (1.85 - 9.29) | 0.39 |
| Constipation | 83 | 3.54 (1.26 - 9.94) | 5.98 (2.44 - 14.68) | 3.96 (1.64 - 9.57) | | 5.67 (2.31 - 13.93) | 0.58 |
| Diarrhea | 83 | 2.88 (1.14 - 7.32) | 1.99 (0.89 - 4.48) | 3.70 (1.67 - 8.21) | | 2.74 (1.22 - 6.17) | 0.84 |
| Financial impact | 83 | 11.73 (3.97 - 34.63) | 3.24 (1.26 - 8.32) | 5.45 (2.16 - 13.76) | | 5.83 (2.27 - 14.99) | 0.55 |
| **EORTC QLQ-BR23** |  |  |  |  | |  |  |
| **Functioning** |  |  |  |  | |  |  |
| Body image | 83 | 39.26 (18.72 - 82.31) | 43.47 (22.83 - 82.78) | 40.36 (21.43 – 76.00) | | 24.53 (12.86 - 46.80) | 0.21 |
| Sexual functioning | 83 | 3.87 (1.49 - 10.02) | 4.15 (1.81 - 9.50) | 5.46 (2.42 - 12.34) | | 2.48 (1.08 - 5.70) | 0.40 |
| Future perspective | 83 | 13.59 (5.39 - 34.25) | 53.29 (23.84 - 19.13) | | 27.46 (12.46 - 60.54) | 8.93 (3.99 – 20.00) | 0.13 |
| **Symptom** |  |  |  |  | |  |  |
| Systematic therapy side  effects | 83 | 28.28 (16.74 - 47.78) | 18.44 (11.69 - 29.10) | 19.07 (12.18 - 29.85) | | 26.15 (16.55 - 41.31) | 0.94 |
| Breast symptoms | 83 | 15.18 (7.10 - 32.45) | 4.79 (2.48 - 9.28) | 7.05 (3.68 - 13.49) | | 9.53 (4.92 - 18.49) | 0.70 |
| Arm symptoms | 83 | 31.44 (15.60 - 63.35) | 18.38 (9.99 - 33.80) | 15.19 (8.35 - 27.64) | | 21.23 (11.52 - 39.11) | 0.44 |
| Upset by hair loss | 54 | 34.30 (9.14 - 128.64) | 18.14 (4.92 - 66.89) | 30.79 (9.22 - 102.84) | | 46.05 (14.06 - 150.86) | 0.52 |

Abbreviations: LS means, least squares means; 95% CI, 95% confidence interval; HRQoL, health-related quality of life; EORTC QLQ-C30, European Organization for Research and Treatment of Cancer Quality of Life Questionnaire Core 30; BR23, breast cancer module 23; AJCC, American Joint Committee on Cancer

^a^ Adjusted for age (year; continuous), body mass index (kg/m^2^; continuous), energy intake (kcal/d; continuous), marital status (married or cohabitation, others), education level (high school or below, college or above), breast cancer stage at diagnosis (I,II,III), physical activity (MET-hr/wk; continuous) and time since surgery (months; continuous)

**Table S8**. **Least squares (LS) means scores^a^ (95% Confidence intervals, CIs) of HRQoL according to quartiles of Western dietary pattern in breast cancer survivors, postmenopausal status at diagnosis**

|  |  | **Quartiles of Western dietary pattern** | | | |  |
| --- | --- | --- | --- | --- | --- | --- |
| **Variables** | **N**  **(=148)** | **Quartile 1** | **Quartile 2** | **Quartile 3** | **Quartile 4** | ***P* for trend** |
| **EORTC QLQ-C30** |  |  |  |  |  |  |
| **Global health status/QoL** | 126 | 30.99 (20.44 - 46.97) | 29.34 (19.23 - 44.78) | 26.39 (16.83 - 41.40) | 48.86 (31.97 - 74.67) | 0.08 |
| **Functioning** |  |  |  |  |  |  |
| Physical Functioning | 145 | 70.55 (65.03 - 76.54) | 77.40 (71.31 – 84.00) | 79.24 (72.98 - 86.03) | 73.98 (67.93 - 80.57) | 0.37 |
| Role Functioning | 146 | 68.77 (55.22 - 85.65) | 74.16 (59.32 - 92.70) | 74.49 (59.26 - 93.61) | 64.08 (50.32 - 81.60) | 0.62 |
| Emotional Functioning | 147 | 69.31 (55.25 - 86.95) | 68.01 (54.03 - 85.60) | 72.71 (57.42 - 92.07) | 62.75 (49.09 - 80.20) | 0.57 |
| Cognitive Functioning | 147 | 76.24 (63.73 - 91.21) | 66.28 (55.26 - 79.51) | 74.81 (62.07 - 90.17) | 77.80 (64.08 - 94.46) | 0.66 |
| Social Functioning | 147 | 65.28 (51.10- 83.40) | 62.28 (48.57 - 79.86) | 62.84 (48.69 - 81.10) | 60.74 (46.60 - 79.18) | 0.67 |
| **Symptom** |  |  |  |  |  |  |
| Fatigue | 146 | 26.14 (17.63 - 38.75) | 23.63 (16.03 - 34.84) | 23.08 (15.48 - 34.39) | 23.66 (15.64 - 35.77) | 0.69 |
| Nausea and vomiting^b^ | 147 | 6.81 (3.98 - 11.64) | 2.73 (1.59 - 4.71) | 2.53 (1.45 - 4.43) | 3.05 (1.71 - 5.45) | 0.03 |
| Pain | 146 | 18.45 (9.91 - 34.35) | 10.61 (5.75 - 19.58) | 13.30 (7.09 - 24.97) | 8.74 (4.55 - 16.79) | 0.10 |
| Dyspnea | 145 | 6.45 (3.31 - 12.58) | 8.58 (4.44 - 16.58) | 5.18 (2.62 - 10.25) | 6.11 (3.03 - 12.31) | 0.68 |
| Insomnia | 145 | 29.63 (15.46 - 56.81) | 10.48 (5.49 – 20.00) | 18.92 (9.78 - 36.59) | 24.62 (12.40 - 48.89) | 0.99 |
| Loss of appetite | 145 | 3.51 (1.82 - 6.79) | 3.37 (1.75 - 6.49) | 3.20 (1.64 - 6.24) | 3.36 (1.68 - 6.73) | 0.90 |
| Constipation | 145 | 6.34 (3.19 - 12.57) | 4.67 (2.38 - 9.16) | 5.37 (2.69 - 10.74) | 12.69 (6.20 - 25.97) | 0.09 |
| Diarrhea | 147 | 2.78 (1.50 - 5.14) | 1.92 (1.03 - 3.59) | 3.08 (1.62 - 5.84) | 2.59 (1.33 - 5.04) | 0.91 |
| Financial impact | 147 | 10.71 (5.48 - 20.94) | 11.73 (5.94 - 23.15) | 8.38 (4.17 - 16.84) | 8.73 (4.23 - 18.03) | 0.52 |
| **EORTC QLQ-BR23** |  |  |  |  |  |  |
| **Functioning** |  |  |  |  |  |  |
| Body image | 146 | 40.42 (25.84 - 63.24) | 26.82 (17.12 - 42.03) | 43.78 (27.62 - 69.38) | 40.20 (24.91 - 64.87) | 0.68 |
| Sexual functioning | 133 | 3.37 (1.71 - 6.64) | 1.44 (0.72 - 2.89) | 3.78 (1.84 - 7.76) | 4.81 (2.28 - 10.15) | 0.17 |
| Future perspective | 146 | 19.81 (10.61 - 36.96) | 15.13 (8.09 - 28.30) | 26.10 (13.74 - 49.60) | 20.05 (10.29 - 39.06) | 0.73 |
| **Symptom** |  |  |  |  |  |  |
| Systematic therapy side  effects | 147 | 24.57 (17.02 - 35.48) | 20.33 (14.00 - 29.51) | 18.00 (12.28 - 26.40) | 22.20 (14.92 - 33.04) | 0.62 |
| Breast symptoms | 147 | 18.41 (10.64 - 31.88) | 9.45 (5.41 - 16.49) | 10.30 (5.82 - 18.24) | 13.18 (7.28 - 23.88) | 0.43 |
| Arm symptoms | 147 | 28.82 (17.64 - 47.09) | 24.56 (14.92 - 40.42) | 28.58 (17.14 - 47.66) | 23.89 (14.04 - 40.64) | 0.64 |
| Upset by hair loss | 97 | 28.74 (12.82 - 64.43) | 43.87 (18.39 - 104.63) | 30.92 (12.02 - 79.53) | 11.90 (4.80 - 29.50) | 0.10 |

Abbreviations: LS means, least squares means; 95% CI, 95% confidence interval; HRQoL, health-related quality of life; EORTC QLQ-C30, European Organization for Research and Treatment of Cancer Quality of Life Questionnaire Core 30; BR23, breast cancer module 23; AJCC, American Joint Committee on Cancer

^a^ Adjusted for age (year; continuous), body mass index (kg/m^2^; continuous), energy intake (kcal/d; continuous), marital status (married or cohabitation, others), education level (high school or below, college or above), breast cancer stage at diagnosis (I,II,III), physical activity (MET-hr/wk; continuous) and time since surgery (months; continuous).

^b^ P value for comparing top with bottom quintiles <0.05

**Table S9**. **Least squares (LS) means scores^a^ (95% Confidence intervals, CIs) of HRQoL according to quartiles of Healthy dietary pattern in breast cancer survivors, time since surgery (< 2 years)**

|  |  | **Quartiles of Healthy dietary pattern** | | | |  |
| --- | --- | --- | --- | --- | --- | --- |
| **Variables** | **N (=116)** | **Quartile 1** | **Quartile 2** | **Quartile 3** | **Quartile 4** | ***P* for trend** |
| **EORTC QLQ-C30** |  |  |  |  |  |  |
| **Global health status/QoL** | 101 | 32.29 (17.87 - 58.33) | 36.05 (22.48 - 57.83) | 36.60 (21.33 - 62.79) | 33.06 (20.27 - 53.93) | 0.97 |
| **Functioning** |  |  |  |  |  |  |
| Physical Functioning | 116 | 72.29 (64.64 - 80.85) | 76.95 (70.33 - 84.20) | 76.11 (68.67 - 84.36) | 77.03 (70.17 - 84.57) | 0.44 |
| Role Functioning | 116 | 48.80 (32.61 - 73.01) | 78.78 (56.97 - 108.94) | 75.18 (51.91 - 108.89) | 70.99 (50.73 - 99.35) | 0.23 |
| Emotional Functioning | 116 | 70.38 (54.86 - 90.29) | 79.98 (65.46 - 97.72) | 66.42 (52.83 - 83.51) | 71.93 (58.44 - 88.54) | 0.81 |
| Cognitive Functioning | 116 | 66.16 (51.71 - 84.64) | 84.37 (69.20 - 102.85) | 77.87 (62.09 - 97.65) | 67.32 (54.81 - 82.67) | 0.81 |
| Social Functioning | 116 | 45.41 (29.44 - 70.05) | 66.45 (46.89 - 94.15) | 63.60 (42.70 - 94.73) | 59.34 (41.34 - 85.18) | 0.44 |
| **Symptom** |  |  |  |  |  |  |
| Fatigue | 116 | 36.13 (22.39 - 58.30) | 16.76 (11.41 - 24.63) | 28.00 (18.04 - 43.47) | 23.83 (15.99 - 35.52) | 0.51 |
| Nausea and vomiting | 116 | 6.42 (3.13 - 13.20) | 1.82 (1.02 - 3.25) | 2.34 (1.21 - 4.54) | 2.77 (1.52 - 5.06) | 0.18 |
| Pain | 116 | 19.42 (9.48 - 39.78) | 7.62 (4.28 - 13.56) | 15.33 (7.93 - 29.64) | 8.82 (4.85 - 16.04) | 0.25 |
| Dyspnea | 115 | 7.18 (3.02 - 17.07) | 3.53 (1.78 – 7.00) | 4.09 (1.87 - 8.97) | 2.86 (1.41 - 5.84) | 0.14 |
| Insomnia | 115 | 19.96 (9.54 - 41.78) | 10.08 (5.56 - 18.29) | 26.21 (13.27 - 51.76) | 33.03 (17.78 - 61.35) | 0.08 |
| Loss of appetite | 115 | 3.95 (1.70 - 9.16) | 2.61 (1.33 - 5.15) | 2.29 (1.05 - 4.97) | 3.64 (1.80 - 7.37) | 0.92 |
| Constipation | 115 | 7.14 (3.00 - 16.96) | 6.17 (3.08 - 12.38) | 6.97 (3.11 - 15.59) | 5.21 (2.53 - 10.74) | 0.60 |
| Diarrhea | 116 | 2.03 (0.96 - 4.30) | 3.31 (1.81 - 6.07) | 3.69 (1.85 - 7.37) | 2.01 (1.08 - 3.77) | 0.90 |
| Financial impact | 116 | 12.29 (4.95 - 30.48) | 4.57 (2.20 - 9.48) | 5.63 (2.44 - 12.97) | 6.02 (2.82 - 12.83) | 0.34 |
| **EORTC QLQ-BR23** |  |  |  |  |  |  |
| **Functioning** |  |  |  |  |  |  |
| Body image | 116 | 39.51 (21.06 - 74.14) | 35.11 (21.16 - 58.23) | 32.20 (18.06 - 57.43) | 25.78 (15.25 - 43.56) | 0.26 |
| Sexual functioning | 107 | 1.91 (0.82 - 4.45) | 4.31 (2.17 - 8.58) | 4.76 (2.28 - 9.94) | 4.46 (2.16 - 9.24) | 0.13 |
| Future perspective | 116 | 18.37 (7.96 - 42.40) | 32.13 (16.40 - 62.96) | 25.17 (11.67 - 54.29) | 15.71 (7.82 - 31.56) | 0.57 |
| **Symptom** |  |  |  |  |  |  |
| Systematic therapy side  effects | 116 | 27.48 (16.82 - 44.89) | 10.64 (7.17 - 15.79) | 22.52 (14.34 - 35.37) | 22.94 (15.23 - 34.55) | 0.71 |
| Breast symptoms | 116 | 17.58 (9.08 - 34.03) | 7.38 (4.34 - 12.55) | 13.95 (7.60 - 25.60) | 9.13 (5.26 - 15.84) | 0.33 |
| Arm symptoms | 116 | 30.46 (16.93 - 54.79) | 20.42 (12.74 - 32.75) | 24.72 (14.41 - 42.41) | 30.42 (18.64 - 49.64) | 0.78 |
| Upset by hair loss^b^ | 70 | 7.00 (2.37 - 20.65) | 32.31 (11.28 - 92.51) | 40.90 (15.65 - 106.90) | 37.74 (15.91 - 89.52) | 0.02 |

Abbreviations: LS means, least squares means; 95% CI, 95% confidence interval; HRQoL, health-related quality of life; EORTC QLQ-C30, European Organization for Research and Treatment of Cancer Quality of Life Questionnaire Core 30; BR23, breast cancer module 23; AJCC, American Joint Committee on Cancer

^a^ Adjusted for age (year; continuous), body mass index (kg/m^2^; continuous), energy intake (kcal/d; continuous), marital status (married or cohabitation, others), education level (high school or below, college or above), breast cancer stage (I,II,III), physical activity (MET-hr/wk; continuous) and menopausal status at diagnosis (premenopausal, postmenopausal status)

^b^ P value for comparing top with bottom quintiles <0.05

**Table S10. Least squares (LS) means scores^a^ (95% Confidence intervals, CIs) of HRQoL according to quartiles of Healthy dietary pattern in breast cancer survivors, time since surgery (≥ 2 years)**

|  |  | **Quartiles of Healthy dietary pattern** | | | |  |
| --- | --- | --- | --- | --- | --- | --- |
| **Variables** | **N (=116)** | **Quartile 1** | **Quartile 2** | **Quartile 3** | **Quartile 4** | ***P* for trend** |
| **EORTC QLQ-C30** |  |  |  |  |  |  |
| **Global health status/QoL** | 99 | 48.06 (30.20 - 76.50) | 42.21 (26.21 - 67.96) | 41.71 (26.79 - 64.93) | 26.96 (14.65 - 49.62) | 0.16 |
| **Functioning** |  |  |  |  |  |  |
| Physical Functioning | 112 | 79.13 (62.86 - 99.62) | 81.51 (64.95 - 102.31) | 79.70 (64.24 - 98.88) | 62.05 (46.87 - 82.16) | 0.28 |
| Role Functioning | 113 | 71.03 (51.74 - 97.51) | 73.82 (52.90 - 103.01) | 64.24 (46.65 - 88.45) | 55.57 (36.54 - 84.50) | 0.30 |
| Emotional Functioning | 114 | 75.30 (55.46 - 102.22) | 58.16 (42.18 - 80.20) | 53.73 (39.60 - 72.89) | 58.95 (39.38 - 88.24) | 0.09 |
| Cognitive Functioning | 114 | 77.35 (66.23 - 90.33) | 70.03 (59.50 - 82.44) | 68.40 (58.59 - 79.85) | 73.54 (59.93 - 90.25) | 0.32 |
| Social Functioning | 114 | 69.03 (53.76 - 88.64) | 58.18 (44.73 - 75.66) | 60.43 (47.09 - 77.55) | 67.75 (48.71 - 94.23) | 0.60 |
| **Symptom** |  |  |  |  |  |  |
| Fatigue | 113 | 23.06 (13.80 - 38.53) | 26.00 (15.43 - 43.81) | 20.11 (12.26 - 32.96) | 22.56 (11.78 - 43.18) | 0.75 |
| Nausea and vomiting | 114 | 3.55 (1.81 - 6.97) | 3.65 (1.79 - 7.41) | 5.96 (3.04 - 11.69) | 5.71 (2.34 - 13.90) | 0.17 |
| Pain | 113 | 5.49 (2.36 - 12.78) | 5.41 (2.29 - 12.77) | 7.70 (3.41 - 17.36) | 14.34 (4.93 - 41.71) | 0.13 |
| Dyspnea^b^ | 113 | 11.77 (5.12 - 27.02) | 6.15 (2.64 - 14.32) | 6.82 (3.06 - 15.19) | 3.14 (1.10 - 8.99) | 0.03 |
| Insomnia^b^ | 113 | 8.01 (3.30 - 19.47) | 18.48 (7.50 - 45.54) | 16.25 (6.91 - 38.18) | 31.01 (10.10 - 95.25) | 0.03 |
| Loss of appetite | 113 | 2.27 (1.03 – 5.00) | 4.44 (1.99 - 9.90) | 2.36 (1.10 - 5.04) | 2.17 (0.80 - 5.90) | 0.94 |
| Constipation | 113 | 2.29 (0.96 - 5.44) | 4.17 (1.73 - 10.04) | 5.36 (2.33 - 12.34) | 2.23 (0.75 - 6.65) | 0.41 |
| Diarrhea | 114 | 3.16 (1.51 - 6.61) | 1.81 (0.83 - 3.93) | 2.42 (1.16 - 5.06) | 1.32 (0.50- 3.50) | 0.16 |
| Financial impact^b^ | 114 | 6.62 (3.01 - 14.57) | 6.12 (2.67 - 14.02) | 18.10 (8.24 - 39.75) | 26.64 (9.41 - 75.38) | 0.01 |
| **EORTC QLQ-BR23** |  |  |  |  |  |  |
| **Functioning** |  |  |  |  |  |  |
| Body image | 113 | 43.08 (25.52 - 72.73) | 33.53 (19.33 - 58.15) | 48.57 (28.57 - 82.58) | 34.34 (17.21 - 68.51) | 0.85 |
| Sexual functioning | 109 | 4.75 (2.12 - 10.63) | 4.56 (2.01 - 10.37) | 4.37 (1.91 - 10.03) | 3.97 (1.43 - 11.01) | 0.75 |
| Future perspective | 113 | 22.70 (11.19 - 46.05) | 8.61 (4.09 - 18.11) | 15.11 (7.38 - 30.95) | 10.84 (4.27 - 27.56) | 0.15 |
| **Symptom** |  |  |  |  |  |  |
| Systematic therapy side  effects | 114 | 25.56 (17.65 - 37.02) | 26.16 (17.72 - 38.61) | 26.17 (18.08 - 37.86) | 35.37 (21.70 - 57.66) | 0.37 |
| Breast symptoms | 114 | 10.46 (5.31 - 20.62) | 9.26 (4.54 - 18.91) | 11.35 (5.77 - 22.33) | 27.68 (11.31 - 67.77) | 0.14 |
| Arm symptoms | 114 | 15.32 (8.14 - 28.83) | 18.21 (9.37 - 35.40) | 21.9 (11.65 - 41.16) | 37.89 (16.45 - 87.28) | 0.06 |
| Upset by hair loss | 81 | 31.11 (10.61 - 91.27) | 46.85 (15.81 - 138.78) | 23.87 (8.97 - 63.52) | 16.18 (4.39 - 59.60) | 0.41 |

Abbreviations: LS means, least squares means; 95% CI, 95% confidence interval; HRQoL, health-related quality of life; EORTC QLQ-C30, European Organization for Research and Treatment of Cancer Quality of Life Questionnaire Core 30; BR23, breast cancer module 23; AJCC, American Joint Committee on Cancer

^a^ Adjusted for age (year; continuous), body mass index (kg/m^2^; continuous), energy intake (kcal/d; continuous), marital status (married or cohabitation, others), education level (high school or below, college or above), breast cancer stage (I,II,III), physical activity (MET-hr/wk; continuous) and menopausal status at diagnosis (premenopausal, postmenopausal status)

^b^ P value for comparing top with bottom quintiles <0.05

**Table S11**. **Least squares (LS) means scores^a^ (95% Confidence intervals, CIs) of HRQoL according to quartiles of Western dietary pattern in breast cancer survivors, time since surgery (< 2 years)**

|  |  | **Quartiles of Western dietary pattern** | | | |  |
| --- | --- | --- | --- | --- | --- | --- |
| **Variables** | **N (=116)** | **Quartile 1** | **Quartile 2** | **Quartile 3** | **Quartile 4** | ***P* for trend** |
| **EORTC QLQ-C30** |  |  |  |  |  |  |
| **Global health status/QoL** | 101 | 38.22 (23.53 - 62.10) | 30.14 (17.92 - 50.69) | 29.05 (17.82 - 47.35) | 41.95 (25.76 - 68.31) | 0.74 |
| **Functioning** |  |  |  |  |  |  |
| Physical Functioning | 116 | 68.78 (62.86 - 75.26) | 78.55 (71.67 - 86.08) | 82.52 (75.43 - 90.28) | 74.17 (67.54 - 81.46) | 0.24 |
| Role Functioning | 116 | 57.98 (41.21 - 81.56) | 73.96 (52.27 - 104.66) | 81.89 (58.24 - 115.12) | 65.77 (46.11 - 93.82) | 0.59 |
| Emotional Functioning | 116 | 72.25 (58.53 - 89.19) | 70.44 (56.85 - 87.27) | 76.74 (62.19 - 94.70) | 71.33 (57.28 - 88.81) | 0.98 |
| Cognitive Functioning | 116 | 78.93 (63.99 - 97.36) | 68.26 (55.14 - 84.50) | 76.88 (62.35 - 94.79) | 73.95 (59.44 - 91.99) | 0.81 |
| Social Functioning | 116 | 61.02 (42.25 - 88.12) | 61.83 (42.54 - 89.85) | 62.02 (42.97 - 89.50) | 53.64 (36.59 - 78.63) | 0.61 |
| **Symptom** |  |  |  |  |  |  |
| Fatigue | 116 | 28.82 (19.04 - 43.61) | 21.71 (14.24 - 33.09) | 23.06 (15.25 - 34.87) | 22.73 (14.77 - 34.98) | 0.46 |
| Nausea and vomiting | 116 | 4.10 (2.23 - 7.54) | 1.78 (0.96 - 3.31) | 1.89 (1.03 - 3.47) | 4.06 (2.16 - 7.64) | 0.90 |
| Pain | 116 | 17.39 (9.45 - 32.03) | 8.99 (4.83 - 16.72) | 9.53 (5.18 - 17.54) | 10.02 (5.31 - 18.91) | 0.24 |
| Dyspnea | 115 | 3.42 (1.69 - 6.92) | 7.66 (3.75 - 15.66) | 2.18 (1.07 - 4.44) | 4.04 (1.94 - 8.40) | 0.83 |
| Insomnia | 115 | 27.99 (14.84 - 52.80) | 11.34 (5.92 - 21.72) | 22.39 (11.9 - 42.15) | 22.25 (11.49 - 43.11) | 0.90 |
| Loss of appetite | 115 | 3.49 (1.72 - 7.07) | 2.04 (0.99 - 4.21) | 2.98 (1.47 - 6.04) | 3.85 (1.84 - 8.06) | 0.69 |
| Constipation | 115 | 4.45 (2.15 - 9.19) | 7.27 (3.49 - 15.13) | 5.22 (2.54 - 10.73) | 8.74 (4.13 - 18.49) | 0.24 |
| Diarrhea | 116 | 2.96 (1.57 - 5.55) | 1.77 (0.93 - 3.36) | 3.80 (2.02 - 7.13) | 2.66 (1.38 - 5.13) | 0.89 |
| Financial impact | 116 | 10.01 (4.65 - 21.54) | 4.98 (2.29 - 10.87) | 5.60 (2.60 - 12.03) | 5.29 (2.38 - 11.75) | 0.28 |
| **EORTC QLQ-BR23** |  |  |  |  |  |  |
| **Functioning** |  |  |  |  |  |  |
| Body image | 116 | 34.25 (20.14 - 58.25) | 33.35 (19.43 - 57.25) | 33.95 (19.98 - 57.68) | 27.57 (15.87 - 47.92) | 0.56 |
| Sexual functioning | 107 | 4.60 (2.37 - 8.94) | 1.91 (0.97 - 3.77) | 7.95 (3.83 - 16.52) | 4.00 (1.92 - 8.33) | 0.75 |
| Future perspective | 116 | 17.81 (8.80 - 36.05) | 28.01 (13.67 - 57.40) | 28.74 (14.21 - 58.10) | 17.33 (8.32 - 36.11) | 0.92 |
| **Symptom** |  |  |  |  |  |  |
| Systematic therapy side  effects | 116 | 24.70 (16.13 - 37.84) | 13.99 (9.07 - 21.59) | 15.18 (9.92 - 23.24) | 23.34 (14.97 - 36.37) | 0.98 |
| Breast symptoms | 116 | 18.12 (10.40 - 31.55) | 8.56 (4.87 - 15.05) | 8.30 (4.77 - 14.44) | 9.96 (5.59 - 17.75) | 0.15 |
| Arm symptoms | 116 | 31.18 (19.11 - 50.87) | 23.21 (14.11 - 38.18) | 32.11 (19.7 - 52.33) | 18.61 (11.18 - 30.98) | 0.20 |
| Upset by hair loss | 70 | 18.95 (7.80 - 46.05) | 47.23 (14.49 - 153.88) | 51.34 (18.75 - 140.55) | 22.82 (8.93 - 58.32) | 0.80 |

Abbreviations: LS means, least squares means; 95% CI, 95% confidence interval; HRQoL, health-related quality of life; EORTC QLQ-C30, European Organization for Research and Treatment of Cancer Quality of Life Questionnaire Core 30; BR23, breast cancer module 23; AJCC, American Joint Committee on Cancer

^a^ Adjusted for age (year; continuous), body mass index (kg/m^2^; continuous), energy intake (kcal/d; continuous), marital status (married or cohabitation, others), education level (high school or below, college or above), breast cancer stage (I,II,III), physical activity (MET-hr/wk; continuous) and menopausal status at diagnosis (premenopausal, postmenopausal status).

**Table S12**. **Least squares (LS) means scores^a^ (95% Confidence intervals, CIs) of HRQoL according to quartiles of Western dietary pattern in breast cancer survivors, time since surgery (≥ 2 years)**

|  |  | | **Quartiles of Western dietary pattern** | | | |  |
| --- | --- | --- | --- | --- | --- | --- | --- |
| **Variables** | **N (=116)** | | **Quartile 1** | **Quartile 2** | **Quartile 3** | **Quartile 4** | ***P* for trend** |
| **EORTC QLQ-C30** |  | |  |  |  |  |  |
| **Global health status/QoL** | 99 | | 30.39 (18.10 - 51.03) | 43.53 (27.57 - 68.73) | 37.12 (22.63 - 60.89) | 47.86 (30.07 - 76.18) | 0.17 |
| **Functioning** |  | |  |  |  |  |  |
| Physical Functioning | 112 | | 65.17 (50.86 - 83.51) | 75.75 (60.19 - 95.32) | 81.36 (64.71 - 102.30) | 80.29 (64.23 - 100.37) | 0.11 |
| Role Functioning | 113 | | 53.87 (38.29 - 75.79) | 63.56 (46.03 - 87.76) | 78.74 (56.85 - 109.05) | 71.42 (51.18 - 99.67) | 0.10 |
| Emotional Functioning | 114 | | 50.29 (35.98 - 70.27) | 65.74 (47.92 - 90.18) | 71.66 (52.09 - 98.58) | 57.77 (41.93 - 79.58) | 0.48 |
| Cognitive Functioning | 114 | | 66.85 (56.47 - 79.13) | 69.81 (59.52 - 81.87) | 79.18 (67.42 - 92.99) | 71.93 (61.20 - 84.53) | 0.29 |
| Social Functioning | 114 | | 66.67 (50.71 - 87.65) | 61.11 (47.19 - 79.13) | 70.42 (54.25 - 91.39) | 57.63 (44.35 - 74.88) | 0.49 |
| **Symptom** |  | |  |  |  |  |  |
| Fatigue | 113 | | 23.07 (13.05 - 40.76) | 21.11 (12.62 - 35.29) | 24.37 (14.41 - 41.20) | 22.75 (13.55 - 38.19) | 0.91 |
| Nausea and vomiting | 114 | | 8.46 (4.10 - 17.45) | 4.22 (2.13 - 8.36) | 3.28 (1.64 - 6.53) | 4.11 (2.05 - 8.23) | 0.08 |
| Pain | 113 | | 9.20 (3.59 - 23.59) | 7.74 (3.31 - 18.11) | 8.20 (3.44 - 19.54) | 4.96 (2.11 - 11.68) | 0.24 |
| Dyspnea | 113 | | 6.92 (2.70 - 17.75) | 7.25 (3.10 - 16.97) | 8.30 (3.48 - 19.80) | 5.68 (2.41 - 13.38) | 0.73 |
| Insomnia | 113 | | 25.08 (9.24 - 68.07) | 11.97 (4.86 - 29.47) | 16.45 (6.55 - 41.32) | 14.19 (5.72 - 35.20) | 0.45 |
| Loss of appetite | 113 | | 2.59 (1.08 - 6.21) | 3.67 (1.66 - 8.08) | 1.79 (0.80 - 4.02) | 3.00 (1.35 - 6.65) | 0.87 |
| Constipation | 113 | | 3.37 (1.29 - 8.86) | 2.31 (0.96 - 5.51) | 3.25 (1.33 - 7.91) | 5.55 (2.31 - 13.37) | 0.22 |
| Diarrhea | 114 | | 2.08 (0.92 - 4.70) | 2.05 (0.95 - 4.43) | 3.11 (1.43 - 6.76) | 1.94 (0.89 - 4.25) | 0.94 |
| Financial impact | 114 | | 13.03 (5.29 - 32.11) | 13.53 (5.77 - 31.72) | 10.41 (4.41 - 24.57) | 7.55 (3.19 - 17.91) | 0.22 |
| **EORTC QLQ-BR23** |  | |  |  |  |  |  |
| **Functioning** |  | |  |  |  |  |  |
| Body image | 113 | | 44.31 (24.99 - 78.56) | 28.27 (16.61 - 48.10) | 57.61 (33.71 - 98.46) | 38.99 (22.76 - 66.80) | 0.78 |
| Sexual functioning | 109 | | 4.09 (1.71 - 9.78) | 3.35 (1.41 - 7.96) | 4.34 (1.91 - 9.87) | 5.58 (2.49 - 12.53) | 0.40 |
| Future perspective | 113 | | 14.01 (6.27 - 31.32) | 13.62 (6.45 - 28.72) | 20.76 (9.78 - 44.07) | 10.60 (4.98 - 22.58) | 0.68 |
| **Symptom** |  | |  |  |  |  |  |
| Systematic therapy side  effects | 114 | | 30.28 (20.22 - 45.35) | 30.98 (21.15 - 45.37) | 26.33 (17.92 - 38.70) | 22.76 (15.46 - 33.51) | 0.14 |
| Breast symptoms | 114 | | 18.34 (8.68 - 38.74) | 8.32 (4.10 - 16.86) | 11.93 (5.85 - 24.32) | 12.30 (6.01 - 25.18) | 0.58 |
| Arm symptoms | 114 | | 26.43 (13.15 - 53.11) | 22.07 (11.41 - 42.66) | 15.00 (7.71 - 29.17) | 20.23 (10.37 - 39.46) | 0.38 |
| Upset by hair loss | 81 | 35.28 (11.62 - 107.10) | | 32.00 (11.72 - 87.41) | 34.81 (11.86 - 102.17) | 15.49 (5.15 - 46.62) | 0.24 |

Abbreviations: LS means, least squares means; 95% CI, 95% confidence interval; HRQoL, health-related quality of life; EORTC QLQ-C30, European Organization for Research and Treatment of Cancer Quality of Life Questionnaire Core 30; BR23, breast cancer module 23; AJCC, American Joint Committee on Cancer

^a^ Adjusted for age (year; continuous), body mass index (kg/m^2^; continuous), energy intake (kcal/d; continuous), marital status (married or cohabitation, others), education level (high school or below, college or above), breast cancer stage (I,II,III), physical activity (MET-hr/wk; continuous) and menopausal status at diagnosis (premenopausal, postmenopausal status)
